# Loss-of-consciousness: sources of GABAergic input to the mesopontine tegmental anesthesia area

**DOI:** 10.3389/fnins.2025.1594984

**Published:** 2025-06-23

**Authors:** Angham Ibraheem, Kristina Vaso, Anne Minert, Shai-Lee Yatziv, Mark Baron, Marshall Devor

**Affiliations:** ^1^Department of Cell and Developmental Biology, Institute of Life Sciences, The Hebrew University of Jerusalem, Jerusalem, Israel; ^2^Center for Research on Pain, The Hebrew University of Jerusalem, Jerusalem, Israel

**Keywords:** anesthesia, arousal, extrasynaptic, GABA_*A*_-R, delta subunit, mesopontine tegmentum, sleep

## Abstract

Exposure of neurons in the brainstem mesopontine tegmental anesthesia area (MPTA) to minute quantities of GABAergic general anesthetics at clinically relevant concentrations is sufficient to induce loss-of-consciousness (LOC), while lesioning this nucleus renders rodents relatively insensitive to these anesthetics delivered systemically. The MPTA thus appears to be a key GABA-receptive target in brain mechanisms of clinical anesthesia. As lesioning the MPTA also affects natural instances of LOC including sleep and fainting, it is of interest to know the source(s) of endogenous GABA present in the MPTA. Here, we used retrograde tracing combined with immunolabeling to locate GABAergic neurons that provide the MPTA with synaptic input. Sources of glycinergic and glutamatergic input were also explored. Abundant GABAergic neurons with axonal projections to the MPTA were found in: (1) deep laminae of the neocortex rostrally, (2) a mesolimbic field ranging from the basal forebrain to the limbic midbrain, and (3) deep cerebellar nuclei and the rostroventromedial medulla (RVM). All three showed ipsilateral predominance. Only modest numbers of glycinergic input neurons were found, mostly in the hindbrain. Glutamatergic sources of MPTA input were mainly in the cortex, the ventral tegmental area and the RVM. The endogenous modulatory input to the MPTA identified here, particularly the GABAergic input, likely plays a significant role in the various natural circumstances that involve LOC. GABAergic anesthetics, in turn, agents that permit pain-free surgery, appear to act by substituting for endogenous GABA in the MPTA and hence co-opting endogenous GABA-receptive brain circuitry related to consciousness and its loss.

## Highlights:

• Sources of GABA release in the MPTA were located using retrograde axonal tracing and immunolabeling.
• GABAergic projection neurons reside in frontal cortex, subcortical mesolimbic field, cerebellum and RVM.
• GABAergic drugs delivered to the MPTA yield general anesthesia permitting pain-free surgery.
• Endogenous GABA released in the MPTA may likewise mediate loss-of-consciousness.
• The GABAergic brain circuitry uncovered may contribute to natural sleep, fainting and hibernation.

## 1 Introduction

Transitioning between wakefulness and unconsciousness is natural in processes such as sleep, syncope (fainting) and hibernation, and is also typical of pathologies such as concussion, epilepsy and coma. It is notable that in each instance loss-of-consciousness (LOC) occurs as a “syndrome,” accompanied by atonia/akinesia, analgesia, amnesia and synchronization of burst discharge across large cortical regions resulting in a slow-wave electroencephalographic (EEG) signature (Bharioke et al., 2022; Brown et al., 2012; Volgushev et al., 2006). The similarity of this syndrome across species and behavioral contexts suggests common drive by dedicated, evolutionarily adaptive brain processes. Sleep for example, is thought to benefit memory consolidation and perhaps waste clearance, while fainting restores cerebral blood-flow by lowering the head. General anesthetic agents, which induce a similar syndrome, appear to act by substituting for an endogenous neurotransmitter(s), GABA in the case of GABAergic anesthetics such as barbiturates and propofol, thereby “hijacking” the sleep-wake mechanism (Baron and Devor, 2023; Hemmings et al., 2019; Scharf and Kelz, 2013). In all instances life-sustaining “housekeeping” functions continue to operate, and sometimes also complex sensory and motor processing. To wit, much of the classical research on signal processing in primary sensory cortices was carried out on anesthetized animals. And during parasomnias (e.g., sleep-walking) individuals sometimes carry of complex tasks such as preparing a sandwich, all the while in an unconscious state (Cataldi et al., 2024; Hubel and Wiesel, 1959; Mountcastle et al., 1957).

The question of where the substitution takes place is not resolved. The classical view, still widely held in clinical circles, is that anesthetics distribute in the vasculature and act on GABA_*A*_-receptors (GABA_*A*_-Rs) in widespread locations in the central nervous system (CNS), suppressing consciousness and memory formation by actions in the cerebral cortex, and inducing “immobility” (atonia and analgesia) by actions in the spinal cord (SC). This patch-wise version of the generalized “wet-blanket hypothesis” provides a rational account of anesthetic induction and is consistent with many experimental observations including non-invasive brain imaging (Alkire et al., 1995; Antognini and Carstens, 1998; Franks and Lieb, 1978; Mashour and and Hudetz, 2018; Jerome et al., 2019). A very different hypothesis, proposed originally in the context of research on sleep (French et al., 1953; Grady et al., 2022; Kinomura et al., 1996; Magni et al., 1959; Moruzzi and Magoun, 1949), holds that anesthetics act at one or more CNS nodes in the brainstem reticular activating system (RAS). This, in turn, accesses the cortex to generate EEG changes, amnesia etc., and the spinal cord to generate atonia and analgesia, by means of dedicated ascending and descending axonal pathways. Both hypotheses, “wet-blanket” and “dedicated pathways,” posit that LOC and the other anesthetic endpoints are effected in numerous distant structures, cortex to cord. The difference is that the “dedicated pathways hypothesis” holds that these distributed targets are accessed by axonal pathways rather than directly, by agonist molecules in the circulatory system. The limited temporal and spatial resolution of current functional imaging methods precludes choosing among these hypotheses (Alkire et al., 1995).

A significant step forward was the identification of a small region within the classical RAS which, when exposed to minute quantities of GABA_*A*_-R agonists, rapidly induces and maintains surgical anesthesia and loss of the righting reflex (LORR) coupled with immobility to strong noxious stimuli. See video: https://www.frontiersin.org/articles/10.3389/fnmol.2023.1197304/full#supplementary-material. This is the mesopontine tegmental anesthesia area (MPTA) (Avigdor et al., 2021; Baron and Devor, 2023; Devor and Zalkind, 2001; Mao et al., 2024; Namjoshi et al., 2009; Voss et al., 2005; Yatziv et al., 2020). A brain-wide survey failed to identify any other locus with these characteristics and, to the best of our knowledge, no such locus has been reported by others. Curiously, in a ventrally adjacent region, the oral segment of the pontine tegmentum (PnO), delivery of GABAergic agents promotes wakefulness. And probably not by chance, the dorsal mesopontine tegmentum is also the consensus location for injury-induced coma in humans (Devor and Zalkind, 2001; Grady et al., 2022; Leung et al., 2014; Flint et al., 2010).

The sharp borders of the effective MPTA microinjection target, the rapid onset of LORR and LOC following microinjection (seconds) and the minute quantities of drug that are sufficient, effectively rule out explanations based on diffusion or vascular transport of the active molecules from the MPTA to the cortex and spinal cord. Further support for the dedicated pathways hypothesis is the observation that cell-selective lesions of the MPTA render animals relatively insensitive to GABAergic anesthetics delivered systemically. Not incidentally, cell-selective MPTA lesions also alter natural sleep-wake cycling and sensitivity to fainting caused by hypercapnia (Lanir-Azaria et al., 2018; Meiri et al., 2016; Minert et al., 2020; Minert and Devor, 2016; Minert et al., 2017).

The cellular and molecular underpinnings of MPTA-induced anesthesia are also being revealed. We recently identified a subpopulation of MPTA “effector-neurons” whose activation (not inhibition) using pharmacogenetic tools proved to be pro-anesthetic. These neurons, however, do not express the appropriate GABA_*A*_-R isoform, receptors that include the δ-subunit (hereinafter GABA_*A*_δ-Rs). Effector-neurons are therefore not candidates as the primary cellular target of GABAergic agonists in the MPTA. Rather, a second subpopulation, MPTA “δ-cells,” does express GABA_*A*_δ-Rs. These observations indicate that δ-cells are the primary cellular target of GABAergic agonists in the MPTA. We speculate that these might activate effector-neurons secondarily, perhaps by disinhibition, leading to sedation and anesthesia (Baron et al., 2022; Baron et al., 2025; Devor et al., 2016).

On this background we propose that, as for GABAergic anesthesia, brain-state transitioning under natural circumstances is largely mediated by GABA released from GABAergic neurons that have synaptic terminals within the MPTA. Glycine, a second inhibitory neurotransmitter present in the brainstem, though more prominent spinal cord, is also a candidate as is glutamate, a ubiquitous excitatory neurotransmitter that might act directly on effector neurons. In the present study we systematically examined the whereabouts of distant neurons capable of delivering GABA, glycine and glutamate into the MPTA. Our strategy was to combine retrograde tracing to mark distant neurons that project into the MPTA with immuno-labeling to identify which of these are GABAergic, glycinergic and glutamatergic. Additional experiments were carried out to identify trajectories of the major input and output pathways of the MPTA.

## 2 Materials and methods

### 2.1 Animals and surgeries

Adult female Wistar-derived Sabra strain rats (250–300 g) were used (Lutsky et al., 1984). Animals were maintained in a specific pathogen free (SPF) facility, housed 1-3 per cage in individually ventilated of cages 34 × 39 × 22 cm. The day: night cycle was 12 h:12 h, with lights on at 7:00 a.m. Room temperature was maintained at 21–23°C, and drinking water and food pellets (Harlan Teklad product 2918, Envigo, Indiana, USA) were available *ad libitum*. Experimental protocols were approved by the Institutional Animal Care and Use Committee of the Life Sciences Institute of the Hebrew University of Jerusalem and followed the guidelines of the United States Public Health Service’s Policy on Humane Care and Use of Laboratory Animals.

#### 2.1.1 Tracer microinjection

Animals were anesthetized using Propofol-Lipuro (1%; B. Braun Medical; Melsungen, Germany) administered in repeated bolus doses of 1.0 ml/kg as needed through a tail vein catheter that was inserted under brief sedation with isoflurane. Alternatively, surgery was carried out under chloral hydrate anesthesia (400 mg/kg, i.p., Merck, Darmstadt, Germany). Rats were then mounted prone in the head-holder of a stereotaxic instrument with head level between bregma and lambda and 2% lidocaine was infused into the scalp. The scalp was then opened on the midline, and unilateral or bilateral burr-holes were made in the skull exposing the dura over the MPTA (coordinates: B –8.5 mm caudal to bregma; L ± 1.3 mm lateral to the midline; Minert et al., 2017). A microinjection pipette was then lowered to the depth coordinate of the MPTA (D –6.3 mm below the dura) and 50 nL of one of two alternative retrograde tracers was delivered. In 4 rats the tracer was fluorogold [(FG) 5% in 0.9% NaCl (saline), Fluorochrome, Denver, CO] and in 5 it was the adeno-associated virus (AAV) pAAVrg-hSyn-hM3D(Gq)-mCherry (Addgene, Watertown, MS; viral preps. RRID:Addgene_50474, in phosphate buffered saline (PBS) + 0.001% Pluronic F-68 buffer + 200 mM NaCl; titer ≥ 7 × 10^12^ vg/mL). This AAV, hereinafter abbreviated as AAVrg-mCherry, is transported retrogradely from axon endings that it infects back to their projection neurons of origin.

Micropipettes were heat-drawn from glass tubing with an inner bore volume of 200 nL per mm (inner diameter 0.5 mm; Sutter Instruments, Novato, CA; cat.# BF100-50-15). Solutions to be microinjected were loaded into the cylindrical portion of the pipette by suction from the tip which had been broken to a diameter of 20-30 μm. The butt of the micropipette was then connected to a length of polyethylene tubing to enable extrusion of solutions from the tip using repetitive 10–20 ms positive air pressure pulses (usually 1 Kg/cm^2^), 1/sec., over ∼2 min. The standard volume ejected was 50 nL although larger amounts were used occasionally as noted. Volume ejected was monitored by observing the fluid meniscus within the pipette bore under optical magnification. The micropipette was left in place for ∼3 min. and then slowly withdrawn. The surgical incision was then closed using 5-0 silk sutures (Ethicon, Somerville, N.J.) and a topical antiseptic powder was applied (Dermatol, bismuth subgallate; Floris, Tradyon, Israel). Finally, an analgesic (Tramal, 20 mg/kg i.p.; 100 mg/2 mL; Grunenthal, Aachen, Germany), a prophylactic antibiotic (Penibrin\Ampicillin, 60 mg in 0.2 mL, i.m., Sandoz GmbH, Kundl, Austria) and sterile saline (1–2 mL, subcutaneous) were administered. Animals were kept warm until awakening and then returned to the SPF facility.

A second surgery was carried out in these animals, 3 days after the first in the FG-injected rats and ∼3 weeks after the first for AAVrg-mCherry-injected rats. Here colchicine (15 μL, 5 mM in saline, Sigma, lot #84F-0193) was injected bilaterally into the lateral ventricles (icv, intracerebroventricularly; coordinates B-0.8 mm, L ± 1.5 mm, D-4.2 mm). Colchicine blocks axonal transport causing the accumulation of GABA in the cell soma, thus enhancing the visibility of GABAergic neurons (Ribak et al., 1978). As relatively few GABAergic neurons were observed in the mesopontine tegmentum in these animals, visibility was further enhanced by additionally microinjecting 300 nL colchicine directly into the MPTA. All rats that received colchicine were perfused 1 day after its delivery. Brains were then prepared for histological analysis.

#### 2.1.2 Pathway trajectories and key neuronal types resident in the MPTA

Four rats were microinjected unilaterally in the MPTA with 50 nL of two pathway tracers mixed in the same pipette: retrograde AAVrg-mCherry and anterograde pAAV8-hSyn-hM3D(Gq)-eGFP (hereinafter termed AAV8-eGFP; both Addgene). After ∼3 weeks for tracer migration and expression these rats were perfused. But unlike those noted above, brains were cut in the sagittal or horizontal planes rather than coronally (frontally), facilitating the visualization of trajectories of ascending and descending axonal pathways. Horizontal sections also aid in appreciating decussating axons, axons that pass from one side of the brain to the other. With similar aims, 4 additional rats were microinjected with 20 or 50 nL AAV8-eGFP and processed to visualize pathway trajectories using light-sheet microscopy in whole-mount preparations after tissue clearance using CLARITY, as described by Chung and Deisseroth (2013) and Refaeli and Goshen (2022). The fluorescent (green) signal was derived exclusively from the viral transcript expressed by infected MPTA neurons. No immuno-processing was used to enhance the signal.

To visualize the relation of axon terminals to key neuronal types resident within the MPTA we marked the later as follows. MPTA δ-cells, the putative GABA-receptive inhibitory interneurons, were marked by immuno-labeling with a selective anti-GABA_*A*_δ-R antibody. MPTA effector-neurons, the neurons which when activated drive anesthetic induction, were marked with the aid of the same DREADD-AAV used in our previous chemogenetic studies in which this cell type was discovered (Baron et al., 2022). Specifically, 6 rats were microinjected with pAAV8-hSyn-hM3D(Gq)-mCherry (Addgene), hereinafter abbreviated as AAV8-mCherry, with perfusion ∼3 weeks later. Expression of mCherry (red) ensured their identification as effectors.

### 2.2 Perfusion and histology

#### 2.2.1 Tissue preparation

At the designated survival time rats were overdosed with anesthetic and perfused transcardially with 0.9% saline followed by 10% neutral 0.1 M PO4 buffered formalin (Sigma-Aldrich; MO, United States; cat.# HT501320; pH 7.3), both at 37°C. Heads were kept overnight in the formalin solution (4°C) and the next day brains and the cervical SC were dissected out and, excepting the CLARITY animals, transferred to 30% sucrose in PBS as cryoprotectant until they sank (1–2 day). Within the next 1–3 days tissue was cut serially on a freezing microtome at 50 μm into 10 bins (500 μm separation), most in the coronal/frontal plane, but 4 as noted, in the horizontal and sagittal planes. Plane alignment was adjusted manually on the microtome to best match that of the rat brain atlas of Paxinos and Watson (1998). Sections were stored in PBS with 0.02% sodium azide (4°C).

#### 2.2.2 Visualizing GABAergic, glycinergic and glutamatergic neurons that project to the MPTA and their presynaptic terminals

We began by immuno-labeling a few sections from bins #2 or 4 that included the MPTA to check the accuracy of the retrograde marker microinjection. In animals in which the microinjection was “on-target” (section 2.3.1) we proceeded to immuno-label bins #1 and #6 in their entirety (spacing 250 μm) using anti-GAD67, an immuno-marker of GABAergic neurons (also known as GAD1). An adjacent series of sections from these rats (bin #5) was immuno-labeled with the glycinergic marker GlyT2, and sections of bin #3 were immuno-labeled with the glutamate marker VGlut3 (spacing 500 μm). VGlut3 is less selective than VGlut2 in the sense of marking glutamatergic neurons that also express other transmitters (Fremeau et al., 2002). However, as our aim was to cast a broad net and capture all neurons that might deliver glutamate to the MPTA irrespective of other transmitters, we chose VGlut3.

The same markers visualized corresponding pre-terminal axons and synaptic endings of such neurons within the MPTA. In addition, an antibody to neuroligand 2 (NL2) was used to mark (mostly inhibitory) GABAergic or glycinergic axon terminals within the MPTA, supplemented with anti-vesicular GABA transporter (VGAT) which is selective for GABAergic input. Anti-NL1 and anti-VGlut3 antibodies were used to mark (excitatory) glutamatergic terminals within the MPTA.

#### 2.2.3 Visualizing axonal pathways of the MPTA

Axonal pathways emanating from the MPTA were marked with GFP expressed in neurons that took up and anterogradely transported AAV8-eGFP (green). Additionally, pathways entering the MPTA were marked with AAVrg-mCherry (red) transported retrogradely. Axons of both types were visible in the brains of animals microinjected with the mixture of AAVrg-mCherry and AAV8-eGFP. Visibility of one or both of these virally expressed markers was frequently enhanced using anti-GFP and anti-mCherry immuno-labeling.

#### 2.2.4 Immuno-labeling

A uniform protocol was used for immuno-labeling with all antibodies (Tables 1, 2). Free-floating sections were washed in PBS (3 × 3 min) followed by antigen retrieval in which sections were incubated in warm PBS (85°C) for 8 min. After allowing 20 min for return toward room temperature (RT) sections were placed for 1 h (RT) in blocking solution consisting of 3% fish gelatin (Sigma; Darmstadt, Germany; G-7765; Lot# 37H1205) in PBS including 0.02% azide with 0.25% triton X-100 (TBS, BDH Laboratory Suppliers, United Kingdom). They were then incubated overnight at RT with continuous rotary agitation in a mix of two primary antibodies, one targeting the retrograde tracer used, FG or mCherry (rabbit host in both cases), and the second targeting one of the neurotransmitters. The incubation solutions were PBS containing alternatively, 1.5% fish gelatin, or bovine serum albumin (BSA, MP Biomedicals, Solon, OH, lot.S6866) with 0.02% azide. The following day sections were again washed (PBS, 4 × 4 min) and then transferred to secondary antibodies, one to visualize the retrograde tracer used, anti-FG or anti-mCherry, and the second targeting one of the 3 neurotransmitters, always using contrasting hosts: mouse, guinea pig, donkey or goat. The primary antibodies used and their dilutions, and the secondary antibodies and associated fluorophores used, are laid out in Tables 1, 2. In general a red or infrared fluorophore was used for retrograde labeling (Cy3 or Cy5) and a green fluorophore (Alexa448) for neurotransmitters. For GlyT2-IR and VGlut3-IR the avidin-biotin reaction was used to visualize the primary antibody. Here sections were incubated in a biotinylated donkey anti-guinea pig IgG followed by additional incubation in streptavidin-Cy2 (green). In all cases incubation lasted 1.5–2.0 h (RT) with continuous rotary agitation. After immuno-labeling was completed, sections were washed once again (4 × 4 min, PBS, RT), mounted on glass slides, air-dried and cover-slipped using immu-mount (Thermo-Fisher Scientific, Franklin, MA).

**TABLE 1:** Primary antibodies used for immuno-histochemical labeling.

Neuronal structure to be marked	Antibody target	Host	Vendor	Catalog number	Dilution
Neurons including axons	Anti-mCherry	Rabbit	Abcam	ab183628	1: 1000
Neurons including axons	Anti-GFP	Rabbit	Invitrogen	A-11122	1: 5000
Neurons	Anti-FG	Rabbit	Merck	AB153-I	1: 1000
GABAergic neurons	Anti-GAD67	Mouse	Merck	MAB5406	1: 1000
MPTA δ-neurons	Anti-GABAaδ-R	Rabbit	Alomone	AGA-014	1: 500
Glutamatergic neurons	Anti-VGlut3	Guinea pig	SySy	135 204	1: 500
GABAergic neurons	anti-VGAT	Guinea pig	Chemicon (Merck)	AB5855	1: 1,000
Axon terminals, glutamatergic	Anti-NL1	Mouse	SySy	129 111	1: 500
	Rabbit	SySy	129 003	1: 500
Axon terminals, GABAergic & glycinergic	Anti-NL2	Mouse	SySy	129 511	1: 500
Glycinergic neurons	Anti-GlyT2	Guinea pig	SySy	272 004	1: 500

**TABLE 2:** Secondary antibodies and fluorophores used for visualization of primary antibodies listed in Table 1.

Primary antibody: anti-	Host of primary antibody	Host of secondary antibody	Vendor	Catalog number	Dilution	Fluoro-phore
GAD67, NL1, NL2	Mouse	Donkey IgG	Abcam	ab150105, RRID:AB_2732856	1:500	Alexa 488
VGlut3, VGAT, GlyT2	Guinea pig	Donkey IgG	Jackson Immuno	706-065-148, RRID:AB_2340451	1:1,000	Strept- avidin Cy2
FG	Rabbit	Goat IgG	Millipore	AP132C RRID:AB_92489	1:300	Cy3
mCherry, FG, GABAaδ-R	Rabbit	Goat IgG	Abcam	ab97077, RRID:AB_10679461	1:500	Cy5
mCherry, GFP, NL1	Rabbit	Donkey IgG	Jackson Immuno	488 711-545-152 RRID:AB_2313584	1:1,000	Alexa 488

As noted above, two key subpopulations of MPTA-resident cells have been identified: 1) “effector-neurons,” neurons which when activated drive anesthetic induction and 2) “δ-cells,” putative GABA-receptive inhibitory interneurons. MPTA effector-neurons were recognized by mCherry (red) expressed following infection by AAV8-mCherry microinjected into the MPTA. This is the same DREADD-AAV used in the initial discovery of effector-neurons. δ-cells were recognized using a primary antibody directed against the δ-subunit of GABA_*A*_-R and an appropriate secondary antibody.

#### 2.2.5 Specificity and selectivity of immuno-labeling

Primary antibodies used were raised against peptides specified on the vendors’ websites including verification for specificity using a variety of methods, including Western blot, peptide quenching, and/or knock-out mice. Beyond that, in our hands, we verified the specificity of the antibodies used by observation of immuno-reactivity in neuronal populations known from the literature to express the corresponding epitopes, and its absence in locations in which the marker is not expressed. For example, we confirmed GAD67-IR in neurons of the DCN and Purkinje cells. The GABA_*A*_-R δ-subunit antibody used selectively labeled cerebellar granule cells and was additionally verified in our hands by quenching with the immunizing peptide and by fluorescence *in situ* hybridization (FISH). It was also verified in knock-out mice by Rudolph et al. (2020). Specificity of secondary antibodies used, and of minimal auto-fluorescence, was assured by the absence of labeling in test sections in which the primary or the secondary antibody was omitted. Finally, the retrograde and anterograde tags, FG, mCherry, and GFP, are exogenous molecules and were visible only at locations at which they were microinjected and transported. For all antibodies and antibody combinations used in this study immuno-labeling appeared to penetrate the entire thickness of the section as evaluated by scanning in the Z-axis.

### 2.3 Image capture and regions of interest (ROIs)

#### 2.3.1 Microinjection sites

**FIGURE 1:**
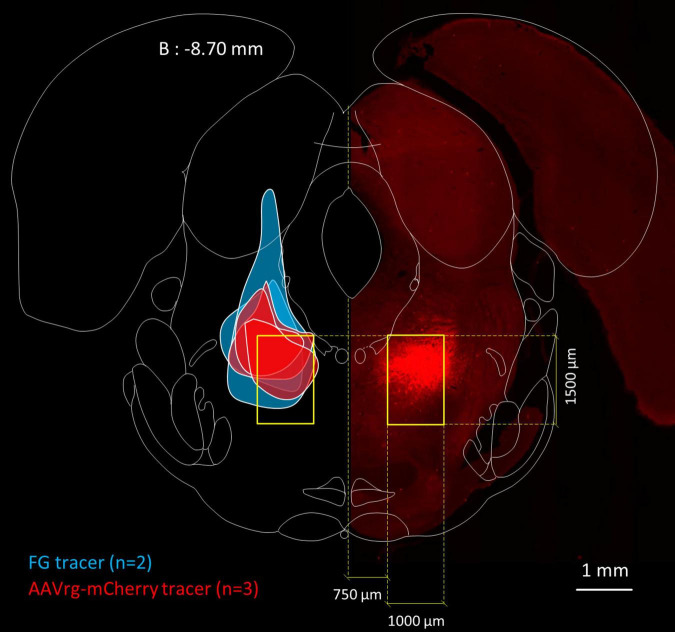
Location of the MPTA and microinjection boundaries. Fluorescence photomicrograph, on the right, showing an MPTA microinjection site expressing mCherry (mirror imaged, rat #AR13), superimposed on a frontal section taken from the rat brain atlas (Paxinos and Watson, 1998), 8.70 mm caudal to bregma (B: –8.70 mm). Yellow rectangles show the location of the MPTA. Colored patches on the left show boundaries of microinjection sites of the 5 animals that contributed to Table 3. From top to bottom (z-axis): 3 rats in which the retrograde tracer was AAVrg-mCherry (red) followed by 2 rats in which the tracer was FG (blue).

The nominal boundaries of the MPTA are defined by a bilaterally symmetrical 1.0 × 1.5 mm rectangle positioned as shown in Figure 1. These are based on the region within which small (10 or 20 nL) microinjections the GABA_*A*_-R agonist muscimol proved to be pro-anesthetic (Minert et al., 2017). Investigators blinded to the experimental results (AI, AM) drew the maximal spread of the injectate in the coronal plane and measured its spread with a digital planimeter (Figure 1). Injection sites were considered to be “on-target” if they covered at least 30% of the 1.5 mm^2^ area of the MPTA (0.45 mm^2^). Using this criterion, 3 of the 8 rats were excluded from the analysis because of off-target microinjections. That left 2 rats in which FG was the retrograde tracer [rats #AR33 (73.1% covered) and AR34 (53.9%)] and 3 in which AAVrg-mCherry was the tracer [rats # AR13 (63.1%), AR14 (30.6%) and AR15 (44.1%), mean 53.0%].

#### 2.3.2 Regions of interest (ROIs)

Whole histological sections extending from the frontal cortex to the cervical SC were scanned as individual images at 40× magnification (4× objective) on a Olympus IX83P2ZF inverted fluorescence microscope fitted with a 2,048 × 2,048 camera (∼ 4.2 megapixel, 16 bit depth) and then stitched using tools in the CellSens Dimentions 3.2 software package. Excitation and emission (barrier) filters were optimized for the fluorophore used (Table 2). Locations that contained numerous retrogradely labeled neurons and ones that were relatively isolated, or of special interest for other reasons were additionally photographed at higher magnification (100×, 10× objective).

**FIGURE 2:**
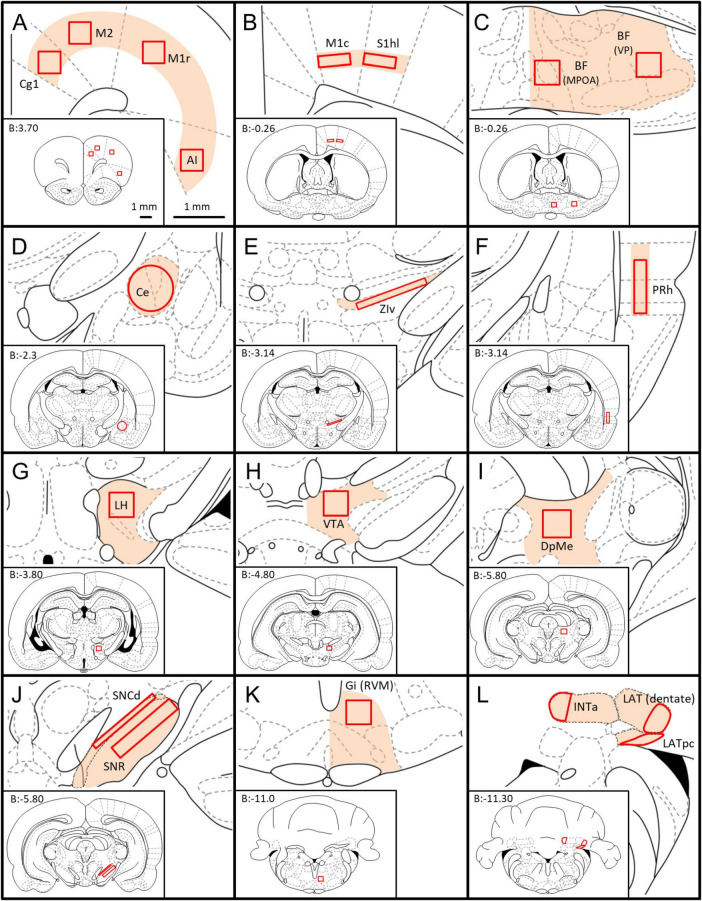
Location of the ROIs used for quantification of GABAergic neurons with axonal projections to the MPTA. Whole coronal brain section outlines, arranged from rostral to caudal **(A–L)**, are shown in the lower left of each panel (Paxinos and Watson, 1998), with enlargement of zones containing ROIs shown in the upper right (light orange fill). The 19 ROIs themselves are indicated by solid red outlines. The scale-bars in **(A)** refer to all panels **(A–L).** **(A)** Cg1—cingulate cortex, area 1; M2—secondary motor cortex; M1r—primary motor cortex, rostral; AI—agranular insular cortex. **(B)** M1c—primary motor cortex, caudal, S1hl—primary somatosensory cortex, hindlimb region. **(C)** BF—basal forebrain [mean of counts in the medial preoptic area (MPOA) and the ventral pallidum (VP)]. **(D)** Ce—central amygdaloid n.; **(E)** ZIv—zona incerta, ventral; **(F)** PRh—perirhinal cortex; **(G)** LH—lateral hypothalamus; **(H)** VTA—ventral tegmental area; **(I)** DpMe—deep mesencephalic n. **(J)** SNCd—susbstantia nigra pars compacta, dorsal; SNR—subtstantia nigra pars reticulate. **(K)** Gi(RVM)—gigantocellular reticular n.; **(L)** INTa—interpositus anterior part of DCN, LAT (dentate)—lateral (dentate) n., LATpc—parvocellular part of lateral n. All 3 together: DCN (deep cerebellar nuclei).

We began by systematically scanning the 40x TIF images of whole sections using Fiji-ImageJ (Schindelin et al., 2012) and manually sketching on corresponding rat atlas sections (Paxinos and Watson, 1998) zones containing substantial numbers of retrogradely labeled neurons and noting their estimated density. These zones were quite consistent in the 5 rats studied (light orange patches in Figure 2). Zones of retrograde labeling were then examined in 100× TIFF images to determine which of them contained even a small fraction of neurons that also expressed GAD67-IR, i.e., neurons that were “double-labeled.” Regions within each zone that had the highest density of double-labeling were then identified, and compared across rats. Finally, we settled on 19 regions at which the density of double-labeled neurons was highest overall, and representative across rats. These 19 served as standard “regions-of-interest” (ROIs; red outlines in Figure 2). One zone was sampled in two sub-regions, medial preoptic area (MPOA) and lateral, ventral pallidum (VP). As values measured were the same across this continuum (*p* > 0.8) an average was made and assigned to a single ROI, the basal forebrain (BF).

The density of double-labeled neurons was consistently higher ipsilateral to the microinjection site than contralateral. For this reason the ROIs were defined on that side and copied to the mirror-symmetrical location contralaterally, even if no double-labeled neurons were actually found contralaterally. We used the 19 standard ROIs selected in this way to quantify the density of retrogradely labeled neurons ipsi- and contralateral to the injection site (neurons per mm^2^), and the proportion that were also GAD67-IR, i.e., double-labeled. Although retrograde labeling was observed in the spinal gray matter (Sukhotinsky et al., 2007) none of these neurons proved to be GAD67-IR. Some, however, expressed GlyT2 or VGlut3.

#### 2.3.3 Cell counting and reconstruction

In each of the 19 ROIs, counts were made of neurons that projected to the MPTA and the proportion that were GABAergic (i.e., also GAD67-IR). ROI nomenclature (see glossary section/abbreviations and Figure 2) followed the rat brain atlas of Paxinos and Watson (1998) with a few deviations. For counting, an optimal 100x image containing the ROI was loaded in Fiji using the color channel appropriate to the retrograde label (red). It was then scanned in a zig-zag manner, upper left to lower right, tallying all red neurons within the ROI with a cursor click, using Fiji’s cell-counter add-in. This marked retrogradely labeled cells on-screen, preventing missed cells or counting the same neuron twice. We then brought up the green channel, representing GAD67-IR neurons, and for each red neuron switched back and forth between red and green channels searching for the presence of both markers at the same location with congruent shape. This was the criterion used to establish neurons as double-labeled. Such neurons sometimes appeared yellow when colors were merged. However, depending on yellow in “merged mode” to determine double-labeling can result in miscounting when the intensity of labeling in one color dominates that of the other. The back-and-forth procedure, used routinely, provided a more reliable estimate of the proportion of projection neurons (red) that were also GABAergic (green). Although we did not use replicate counting by independent investigators in the present study, we did so in previous studies using the same alignment and counting method and obtained concordance values between 1.1% and 3.4% (Goldenberg et al., 2018).

Density of GABAergic projection neurons in each ROI was obtained by dividing the count of double-labeled neurons within the ROI by the area of the ROI in question (neurons per mm^2^, Table 3). Densities given are “nominal” as we did not use stereological or other corrections for cell splitting. Our analysis focused on comparisons across ROIs rather than precision determination of absolute numbers.

**TABLE 3:** Neurons that project to the MPTA (retrogradely labeled): GABAergic and non-GABAergic.

	Density ipsilateral (cells/mm^2^ ± SD)			Density contralateral (cells/mm^2^ ± SD)		
Brain structure: ROI* number of rats (ipsi, contra)	non-GABAergic (single-labeled)	GABAergic (double-labeled)	% double labeled ipsilateral	non-GABAergic (single-labeled)	GABAergic (double-labeled)	% double labeled contralateral
Cg1 (3,3)	520.6 ± 35.6	293.9 ± 121.4	**56.5**	192.9 ± 29.6	67.3 ± 33.3	**34.9**
M2 (4,3)	362.2 ± 190.6	171.3 ± 67.0	**47.3**	60.9 ± 25.4	20.8 ± 20.4	**34.1**
M1r (4,3)	411.5 ± 134.6	216.5 ± 111.0	**52.6**	12.7 ± 11.1	12.7 ± 11.1	**100**
AI (4,3)	605.4 ± 137.8	388.6 ± 106.2	**64.2**	56.7 ± 49.2	38.1 ± 33.2	**67.2**
M1c (5,5)	127.6 ± 82.2	59.7 ± 35.7	**46.8**	9.6 ± 16.4	1.9 ± 4.2	**19.8**
S1hl (5,5)	180.8 ± 65.1	89.0 ± 32.1	**49.2**	17.1 ± 13.9	10.0 ± 12.3	**58.2**
PRh (4,3)	298.5 ± 258.8	137.4 ± 110.1	**46.0**	74.5 ± 129.0	32.4 ± 56.1	**43.5**
**Mean ± SD n = 7**	**358.1 ± 129.2**	**193.8 ± 83.4**	**51.8 ± 6.6**	**60.6 ± 39.2**	**26.2 ± 24.4**	**51.1 ± 26.7**
BF (5,4)	151.2 ± 23.5	12.0 ± 9.1	**8.0**	39.9 ± 24.5	6.2 ± 6.3	**15.6**
Ce (5,5)	500.1 ± 139.6	153.0 ± 36.4	**30.6**	56.3 ± 34.7	13.8 ± 9.7	**24.5**
ZIv (5,5)	322.4 ± 38.4	57.8 ± 19.8	**17.9**	75.0 ± 15.9	10.0 ± 6.6	**13.3**
LH (5,5)	260.9 ± 57.8	39.6 ± 11.1	**15.2**	70.0 ± 18.1	13.8 ± 14.7	**19.6**
VTA (5,5)	232.0 ± 66.9	43.1 ± 14.9	**18.6**	106.2 ± 51.7	18.3 ± 10.2	**17.3**
DpMe (5,5)	345.1 ± 54.9	44.3 ± 20.9	**12.8**	223.1 ± 74.4	21.3 ± 11.0	**9.6**
SNR (5,5)	317.2 ± 55.5	73.2 ± 23.5	**23.1**	170.7 ± 95.3	26.6 ± 18.6	**15.6**
SNCd (5,5)	418.8 ± 112.6	80.6 ± 43.9	**19.3**	236.8 ± 98.1	35.8 ± 21.9	**15.1**
**Mean ± SD n = 8**	**318.5 ± 68.7**	**62.1 ± 41.4**	**18.2 ± 6.8**	**122.2 ± 51.6**	**18.2 ± 12.4**	**16.3 ± 4.4**
Gi (RVM) (4,4)	156.3 ± 27.0	30.6 ± 18.6	**19.6**	71.4 ± 16.8	6.9 ± 8.8	**9.7**
INTa (4,4)	239.2 ± 2.9	136.8 ± 21.8	**57.2**	37.1 ± 0.8	12.5 ± 4.5	**33.6**
LAT (dentate) (4,4)	314.0 ± 2.7	160.8 ± 8.3	**51.2**	75.1 ± 25.2	30.0 ± 5.5	**40.0**
LATpc (4,4)	132.0 ± 8.1	51.9 ± 4.2	**39.3**	49.0 ± 4.6	18.8 ± 0.8	**38.4**
**Mean ± SD n = 4**	**210.4 ± 10.1**	**95.0 ± 13.2**	**41.8 ± 16.6**	**58.1 ± 11.8**	**17.1 ± 4.9**	**30.4 ± 14.1**
**Mean ± SD n = 19**	**310.3 ± 78.7**	**117.9 ± 42.9**	**35.5 ± 18.0**	**86.1 ± 38.7**	**20.9 ± 15.2**	**32.1 ± 23.0**

*ROI names are expanded in the glossary/abbreviations section and in Figure 2. Values in bold indicate percentages and column means.

**FIGURE 3:**
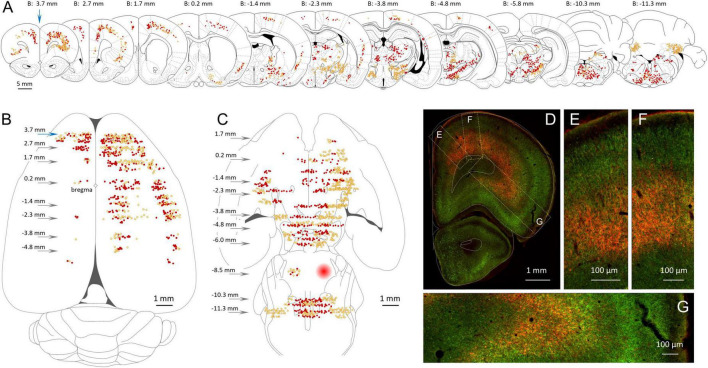
Location of GABAergic neurons with an axonal projection to the MPTA. **(A)** Serial coronal sections taken from the rat brain atlas of Paxinos and Watson (1998) showing the relative density of neurons that project to the MPTA based on retrograde labeling (red, dark dots) and those projection neurons that were GABAergic based on double retrograde and anti-GAD67 immuno-labeling (yellow, light dots). **(B)** Location of the cortical neurons shown in **(A)**, re-plotted on a transparent dorsal view of the cerebral hemispheres. Arrows indicate location of coronal sections with respect to bregma. **(C)** Like **(B)**, but plotting subcortical GABAergic (yellow, light dots) and non-GABAergic projection neurons (red, dark dots). The location of the MPTA microinjection site is indicated by the red circle in the rostral pons (**B:** –8.5 mm). Commissural neurons that project into the microinjection site are indicated by red and yellow dots. **(D–G)** Fluorescence photomicrograph of the far left coronal section in **(A)** (blue arrow, **B:** 3.7 mm). Green fluorescence (GAD67) indicates GABAergic neurons. Red fluorescence (mCherry) indicates retrograde labeling from the MPTA. Many of the GABAergic projection neurons (double-labeled) appear orange or yellow. Rectangular areas (dashed yellow) in **(D)** show the laminar distribution of projection and GABAergic neurons, enlarged in **(E–G)**. Plots and images in **(A–G)** are nearly all from a single animal (#AR15). Documentation of double immuno-labeling is given in Figure 6.

In addition, semi-quantitative evaluation was made of glycinergic (GlyT2-IR) and glutamatergic (VGlu3-IR) projection neurons (Tables 4, 5). Here, the density and proportion double-labeled was estimated subjectively on a scale ranging from dense (+++, comparable to the value in regions with the highest density for the marker in question), to medium (++), sparse (+) and no visible double-labeling (–). Rather than basing these estimates on the (rather small) ROIs, the area evaluated was the entire zone surrounding each ROI (light orange areas in Figure 2). In one case the zone was sampled in 4 ROIs (Figure 2A). Here, parcelation followed the Paxinos and Watson atlas. To aid comparison, this same evaluation routine was applied also to GABAergic neurons (Table 6). Finally, we generated transparent dorsal-view reconstructions of the location of projection neurons, GABAergic (double-labeled, yellow) and non-GABAergic (red), by aligning coronal sections in rostral-to-caudal order and plotting the medio-lateral location of these neurons for the cerebral cortex and separately, for the subcortex (Figures 3B,C).

**TABLE 4:** Neurons that project to the MPTA (retrogradely labeled): glycinergic and non-glycinergic.

Brain structure: ROI* (number of rats)	Non-glycinergic (single labeled) ipsi	Glycinergic (double labeled) ipsi	Non-glycinergic (single labeled) contra	Glycinergic (double labeled) contra
Cg1 (3)	+++	–	+	–
M2 (3)	+++	–	+	–
M1r (3)	+++	–	+	–
AI (3)	+++	–	+	–
M1c (3)	+	++	+	+
S1hl (3)	+	+	+	–
PRh (3)	++	+	+	–
BF (4)	+	+	+	–
Ce (2)	+++	+	+	–
ZIV (3)	++	–	+	–
LH (3)	+++	+	+	+
VTA (3)	++	+	+	–
DpMe (3)	+++	+	++	+
SNR (3)	++	++	+	+
SNCd (3)	++	+	+	+
Gi (RVM) (4)	++	++	+	+
INTa (3)	+++	+	+	+
LAT (dentate) (3)	+++	+	++	+
LATpc (3)	+	+	+	–
SC (3)	+	–	+	–

*ROI names are expanded in the glossary/abbreviations section and in Figure 2. Symbols indicate range: dense (+++), medium (++), sparse (+), no visible labeling (–).

**TABLE 5:** Neurons that project to the MPTA (retrogradely labeled): glutamatergic and non-glutamatergic.

Brain structure: ROI* (number of rats)	Non-glutamatergic (single labeled) ipsi	Glutamatergic (double labeled) ipsi	Non-glutamatergic (single labeled) contra	Glutamatergic (double labeled) contra
Cg1 (3)	+++	+	+	+
M2 (3)	+++	++	+	++
M1r (3)	+++	+++	+	++
AI (3)	+++	+++	+	+
M1c (2)	+	++	–	–
S1hl (2)	+	++	+	–
PRh (2)	+	++	+	++
BF (3)	+	+	+	–
Ce (3)	+++	+	+	–
ZIV (5)	+++	+	+	+
LH (3)	+++	+	+	+
VTA (4)	++	++	+	+
DpMe (4)	+++	+	+	+
SNR (3)	+++	+	+	++
SNCd (2)	++	+	+	+
Gi (RVM) (3)	++	++	++	++
INTa (3)	+++	++	+	+
LAT (dentate) (3)	+++	+	+	+
LATpc (3)	++	+	+	+
SC (3)	+	+	+	+

*ROI names are expanded in the glossary/abbreviations section and in Figure 2. Symbols indicate range: dense (+ + +), medium (+ +), sparse (+), no visible labeling (–).

**TABLE 6:** Neurons that project to the MPTA (retrogradely labeled): GABAergic and non-GABAergic.

Brain structure: ROI* (number of rats)	Non-GABAergic (single labeled) ipsi	GABAergic (double labeled) ipsi	Non-GABAergic (single labeled) contra	GABAergic (double labeled) contra
Cg1 (3)	+++	+++	+	++
M2 (4)	+++	+++	+	++
M1r (4)	+++	+++	+	++
AI (3)	+++	+++	+	+++
M1c (3)	++	+++	+	+++
S1hl (3)	++	+++	+	++
PRh (4)	++	+++	+	+
BF (4)	+	+	+	+
Ce (4)	+++	++	+	+
ZIV (4)	+++	+	+	+
LH (4)	+++	+	+	+
VTA (4)	+++	+	++	+
DpMe (4)	+++	+	+++	+
SNR (4)	+++	++	++	+
SNCd (4)	+++	+	++	+
Gi (RVM) (4)	++	+	+	+
INTa (4)	+++	+++	+	++
LAT (denate) (4)	+++	+++	++	+++
LATpc (4)	++	++	+	++
SC (3)	+	–	+	–

*ROI names are expanded in the glossary/abbreviations section and in Figure 2. Symbols indicate range: dense (+++), medium (++), sparse (+), no visible labeling (–).

#### 2.3.4 Additional microscopic imaging

To attack experimental questions that required higher spatial resolution, notably the relation of afferent axon terminals to postsynaptic neurons, we made confocal image stacks using a Fluoview FV3000 confocal scan head mounted on the Olympus IX83P2ZF microscope (20x air objective, NA = 0.75) using the FV31S-SW software package. A step size of 1 μm was used. Rat brains rendered transparent using CLARITY were imaged using a La-vision Light-Sheet ultra-microscope II (Miltenyi Biotec B.V. & Co., Bielefeld and Göttingen, Germany) running ImspectorPro software.

### 2.4 Statistical evaluation

Nominal density of GABAergic neurons that project to the MPTA in each rat was averaged across rats. No outlier values were excluded, although values for several ROIs were based on counts from fewer than 5 rats due to damaged or missing sections (Table 3). Means and quotients were generally calculated to 4 decimal place accuracy, the values used for statistical analysis. They were then rounded to an accuracy appropriate to the original measurements. Statistical analyses of percentages and densities of neuronal populations were carried out using 2-tailed Student’s *t*-tests with homogeneous or heterogeneous variance, as appropriate. The statistical significance of correlations was tested using the Pearson R-statistic. The software used was Socscistatistics.com and Microsoft Excel. Mean values are given ± the standard deviation (SD). The significance criterion used throughout was *p* ≤ 0.05.

## 3 Results

### 3.1 General findings

**FIGURE 4:**
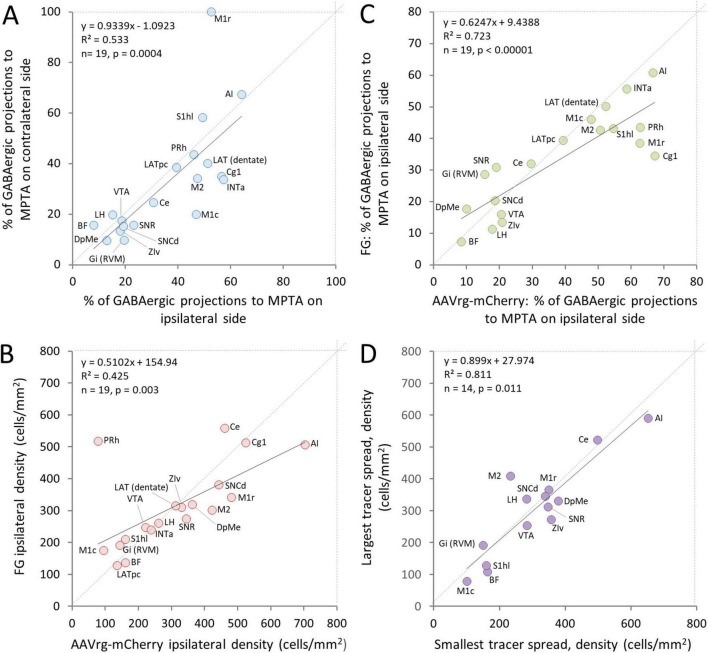
Concordance of three parameters related to neurons with an axonal projection to the MPTA. **(A)** The proportion of neurons across ROIs that have an axonal projection to the MPTA and are GABAergic (double-labeled) is similar for neurons located ipsilateral and contralateral to the microinjection site. **(B)** The density of neurons (not necessarily GABAergic) that had an axonal projection to the MPTA as visualized using FG versus AAVrg-mCherry as retrograde tracers was correlated across ROIs. However, AAVrg-mCherry proved to be more sensitive retrograde tracer. **(C)** The proportion of GABAergic neurons that had an axonal projection to the MPTA as visualized using FG versus AAVrg-mCherry as retrograde tracers was highly correlated, but AAVrg-mCherry proved to be somewhat more robust as a retrograde tracer. Expansion of ROI names is provided in the legend to Figure 2 and in the glossary section. **(D)** The density of neurons projecting into the MPTA in the rat with least spread of tracer beyond its boundaries (#AR13) was similar across ROIs to that of the rat with the greatest tracer spread (#AR33). The one exceptional ROI, PRh, was excluded from the plot (see Discussion section 4).

GABAergic and glutamatergic neurons are located throughout the CNS, albeit with regions of particularly high and low density. Glycinergic neurons, in contrast, are rare in the cortex and relatively infrequent also in the brainstem (Tohyama and Takatsuji, 1998). All of the zones examined except for the spinal cord contained at least some GABAergic and glutamatergic neurons that project to the MPTA, and most had at least a few such glycinergic neurons (Tables 3–6). Densities varied greatly across neurotransmitters, however, and from ROI to ROI. A general finding was lateral asymmetry of projections with labeling density higher ipsilaterally than contralaterally in all 19 ROIs. Overall, ipsi/contra ratio was 6.5 for neurons projecting to the MPTA and 7.3 for GABAergic projection neurons, with the proportion of the latter similar across ROIs (data from all 5 rats; *R*^2^ = 0.533, *p* = 0.0004; Figure 4A). The SC was excluded from this tally. In the 2 rats in which FG was used as retrograde tracer the microinjection was on the right in one and on the left in the other (rats #AR33 and#AR34). Based on the 19 data points in each of these 2 animals, there appears to be bilateral symmetry (*R*^2^ = 0.665, *p* < 0.001). Microinjections in which AAVrg-mCherry was used as tracer were all on the left.

The different mechanisms of cellular uptake and transport of FG and AAVrg-mCherry (Discussion section 4.1) led us to anticipate that cell populations marked with these two tracers might show substantial differences from location to location and in overall transport efficiency. This proved to be only partly so. Re location, regression analysis showed good agreement using the 2 tracers. Densities of retrogradely labeled projection neurons correlated significantly (ipsi *R*^2^ = 0.425, *p* = 0.003; contra *R*^2^ = 0.387, *p* = 0.004, Figure 4B) although 2 of the ROIs, perirhinal cortex (PRh) and Ce, appear to be outliers. Concordance across ROIs in the sub-population of GABAergic MPTA-projection neurons was even more pronounced (ipsi *R*^2^ = 0.723, *p* < 0.0001; contra *R*^2^ = 0.358, *p* = 0.007, Figure 4C). In both cases, however, the slopes of the regression lines were less than one (0.51 and 0.62 respectively, Figures 4B,C) indicating that AAVrg-mCherry is the more efficient tracer. This difference was largely due to several cortical ROIs that innervate the MPTA densely, particularly PRh, primary motor cortex (M1), cingulate cortex area 1 (Cg1) and primary somatosensory cortex, hindlimb region (S1hl).

### 3.2 Location of neurons that project to the MPTA that are GABAergic, glycinergic and glutamatergic: overview

The density of neurons in each of the 19 ROIs that project to the MPTA and the percent that were GABAergic (double-labeled) are shown in Table 3. The ROIs are ordered in the table (roughly) from rostral to caudal. Locations of the ROIs themselves are shown in Figure 2. Overall, density of projection neurons was variable across ROIs, especially ipsilateral to the tracer microinjection site (ipsi range 12.0–388.6 neurons/mm^2^; mean 117.9 ± 42.9; contra range 1.9–67.3 neurons/mm^2^; mean 20.9 ± 15.2).

#### 3.2.1 GABAergic projection neurons

**FIGURE 5:**
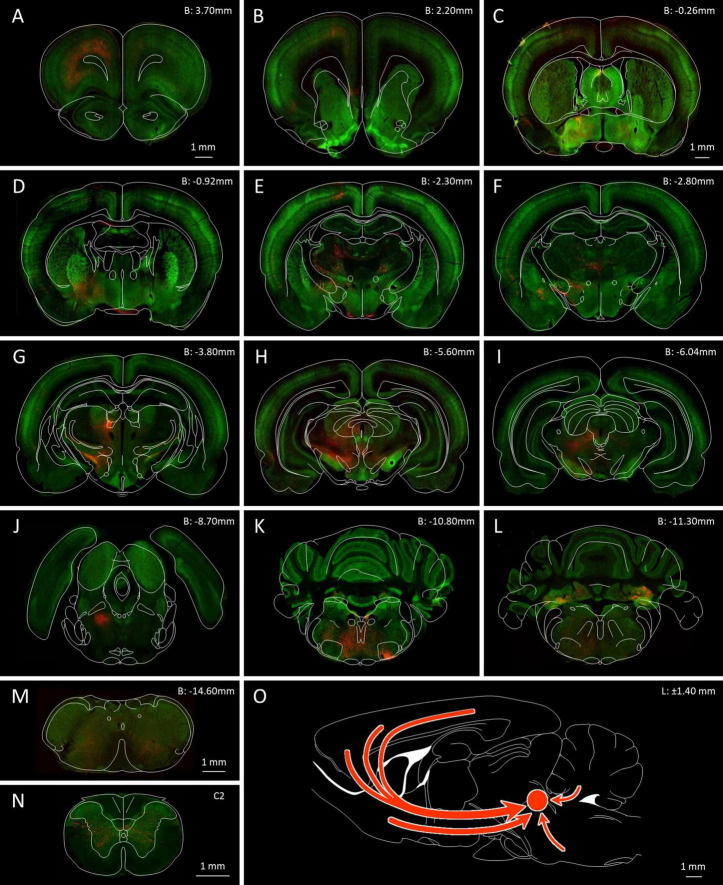
GABAergic neurons in the rat brain and neurons that send an axonal projection to the MPTA. **(A–N)** Serial coronal sections ranging from the PFC (B: 3.70 mm) to the C2 spinal cord, highlighting the location of GABAergic neurons (green fluorescence) and projection neurons retrogradely labeled following AAVrg-mCherry microinjection into MPTA (red fluorescence). The microinjection site in the MPTA is visible in **(J)** (B: –8.70 mm, left side). The scale-bar in **(A)** refers to panels **(A,B)**: The scale-bar in **(C)** refers to panels **(C–L)**. Outline drawings indicating structural parcellation, taken from the Paxinos and Watson atlas, have been superimposed. Red arrows in **(O)** indicate trajectories of the main projections of GABAergic axons that terminate in the MPTA.

Three broad zones contained the bulk of the GABAergic projection neurons. ROIs in the rostral 2/3 of the neocortex constituted the richest source both in terms of density of neurons projecting to the MPTA, the proportion that were GABAergic (double-labeled) and the overall tissue volume containing such neurons (Figures 3, 5). Specifically, the density of GABAergic projection neurons was high (ipsi 193.8 ± 83.4/mm^2^, contralateral 26.2 ± 24.4/mm^2^), with a remarkable half of all MPTA-projection neurons being GABAergic (ipsi mean 51.8 ± 66.0% based of 306 neurons counted in the 7 neocortical ROIs; contra mean 51.1 ± 26.7%). The second broad zone of GABAergic input to the MPTA was a sub-cortical mesolimbic continuum extending from BF rostrally and extending caudally into the lateral hypothalamus (LH), VTA and the substantia nigra (SN). Many fewer GABAergic projection neurons were present in these 8 ROIs than in the rostral cortex (ipsi 61.1 ± 41.4/mm^2^, contra 18.2 ± 12.4/mm^2^), the percent GABAergic was lower (Table 3) and the composite tissue volume was smaller. The third zone was medullary, but selectively so. It included only 4 ROIs: RVM, represented by an ROI in the midline gigantocellular nucleus (Gi), and 3 deep cerebellar nuclei. The density of GABAergic projection neurons was intermediate between the 1st and 2nd zones (ipsi 117.9 ± 42.9/mm^2^, contra 17.1 ± 4.9/mm^2^) as was the percent of projection neurons that were GABAergic (Table 3). The composite tissue volume of this 3rd zone was considerably smaller than that of the other two. Trajectories of these pathways are illustrated in Figure 5O.

Beyond these 3 zones, GABAergic MPTA projection neurons were few or absent, including in the occipital, pyriform and hippocampal cortex, the corpus striatum, thalamus and colliculi. Such neurons were also infrequent in the pons, most of the medulla and the spinal cord. Despite variable density of GABAergic projection neurons across zones and ROIs, the fraction of inputs to the MPTA that were GABAergic was remarkably uniform ipsilaterally and contralaterally in all 3 zones, and surprisingly high: cortex 51.8 and 51.1%, mesolimbic 18.2 and 16.3%, and medulla 41.8 and 30.4%. The overall average was one third (35.5 and 32.1%; Table 3).

#### 3.2.2 Glycinergic and glutamatergic projection neurons

**FIGURE 6:**
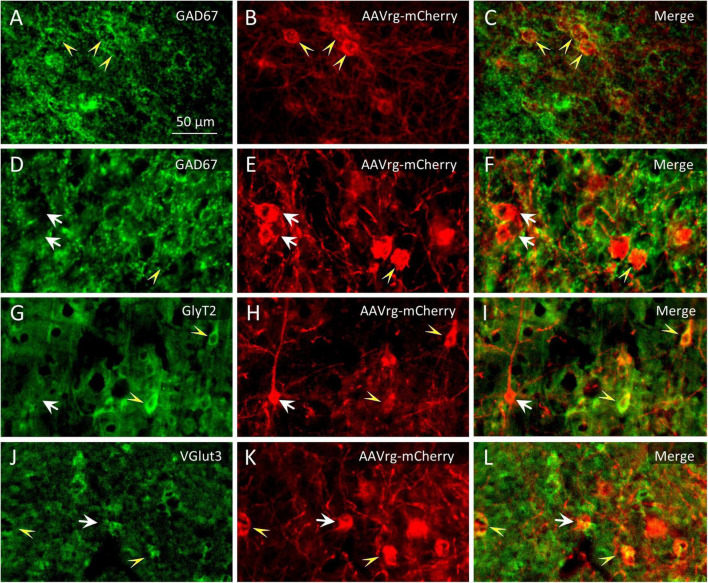
Identification of double immuno-labeled neurons. **(A–F)** Among the neurons that were retrogradely labeled following microinjection of AAVrg-mCherry into the MPTA [red immuno-fluorescence in **(B,E)]** some were GABAergic (GAD67-IR, yellow arrowheads and some were not, white arrows). (**G–I)** Likewise for glycinergic neurons (GlyT2-IR) and (**J–L)** for glutamatergic neurons (VGlut3-IR). The scale-bar in **(A)** refers to all panels **(A–L)**.

Glycinergic neurons were infrequent in neocortical ROIs and relatively few also subcortically. The far rostral cortex was virtually devoid of glycinergic MPTA-projection neurons on either side, although some were encountered further caudally, in the ipsilateral M1 cortex. Subcortically glycinergic neurons were more abundant, but ones with projections to the MPTA remained rare. An exception was the substantia nigra pars reticulata (SNR) and Gi (RVM) in which they were present in modest numbers (Figures 6G–I). Finally, few of the GlyT2-IR neurons in the pons, cerebellum or medulla sent projections to the MPTA (Table 4). Glutamatergic neurons, usually excitatory, were abundant in the cortex with a fair fraction of MPTA-projection neurons showing VGlut3-IR, particularly in the motor cortex ROIs and agranular insular cortex (AI; +++). A notable exception was Cg1 where few double-labeled neurons were seen. There were also many glutamatergic neurons subcortically, in the mesolimbic zone and in the hindbrain, but far fewer of these were MPTA-projection neurons. A modest number of double-labeled neurons was observed in the VTA, the Gi(RVM) and the INTa (Table 5; Figure 6). To facilitate comparison with GABAergic projection neurons, a presentation of GABA input using the qualitative criteria applied to Tables 4, 5 is provided in Table 6.

### 3.3 MPTA projections of GABAergic neurons by zone

#### 3.3.1 Cerebral cortex

The cerebral cortex makes by far the largest contribution of GABAergic input to the MPTA. Ipsilateral and contralateral to the microinjection site double-labeled neurons were located primarily in cortical laminae 5 and 6. Most (∼80%) were large pyramidal neurons (Figures 3C–F). Given the powerful influence that activation of MPTA effector-neurons has on cortical function, EEG, c-FOS expression etc. (Abulafia et al., 2009; Avigdor et al., 2021) this reciprocal relation is noteworthy. Figures 3A,B shows the cortical zone containing GABAergic neurons, including a dorsal view reconstruction of those that project to the MPTA. The cortical ROIs with the highest density of GABAergic projection neurons were in the ipsilateral prefrontal cortex (PFC, ROIs at B + 3.7 mm, blue arrows). These were located especially in Cg1 (ipsi 293.9 ± 121.4/mm^2^), the M1r (ipsi 216.5 ± 111.0/mm^2^) and the AI (ipsi 388.6 ± 106.2/mm^2^). Moving caudally GABAergic projection neurons persisted, becoming gradually less common on the dorsal convexity and the insular region until near the parietal-occipital border. On the contralateral side GABAergic projection neurons were present mostly in the PFC. Moving caudally such neurons became infrequent and only appeared on the dorsal convexity.

#### 3.3.2 Mesolimbic zone

This complex, variegated region controls neuroendocrine functioning via the hypophysis, and high-level regulation of the key homeostatic functions that are associated with motivated behaviors (e.g., feeding and drinking), emotions (e.g., fear, affiliation) and arousal (wake and sleep). It also contributes ascending signals to the cerebral cortex largely via the zona incerta (ZI) and BF as well as descending signals to the pons, medulla and spinal cord (Figures 3A,C, 5C–I; Janig, 2022; Pert, 1997; Stuber and Wise, 2016). The rostral extent of this zone, the fundus striati [ventral striatum, ventral pallidum (VP)] and the adjacent POA and amygdala, are not uniformly endowed with GABAergic MPTA-projections neurons, but rather contain patches of such neurons. In the amygdala, for example, only the Ce contained numerous GABAergic MPTA-projection neurons, comparable in density to the frontal cortex. Many fewer appear elsewhere in the amygdaloid complex. No GABAergic MPTA-projection neurons appeared rostral to the anterior commissure including the olfactory tubercle which is heavily populated with GABAergic neurons. Likewise, neither the corpus striatum, the septum, nor the thalamus contained GABAergic MPTA-projection neurons although some non-GABAergic MPTA-projection neurons were present here. Medial to Ce lies the ventrolateral preoptic nucleus (VLPO), a master regulator of sleep-wake transitions (Lu et al., 2006; Sherin et al., 1996). Although the lateral POA, including VLPO, has direct projections to the MPTA and receives reciprocal projections from the MPTA (Sukhotinsky et al., 2007), we did not encounter any that were GABAergic. Nucleus accumbens, another major limbic affect/arousal node, also appears to have no GABAergic association with the MPTA.

Proceeding caudally, the ZI and adjacent LH, including the perifornical seat of the sleep-related orexinergic neurons, contained relatively few GABAergic MPTA-projection neurons. The same held for the medial hypothalamus and mammillary region, despite the presence there of the histaminergic tuberomammilary nucleus (TMN), also strongly implicated in arousal and sedation (Zecharia et al., 2012). The limbic midbrain likewise contained few GABAergic MPTA-projecting neurons including the VTA and the deep mesencephalic nucleus (DpMe). Considering this trend it was surprising to find both substrantia nigra components, SNR and SNCd, extrapyramidal motor structures with important dopaminergic projections to the striatum, also providing generous GABAergic input to the MPTA. Very few neurons laterally adjacent to the mesolimbic field had axonal projections to the MPTA, GABAergic or otherwise. This includes the thalamic lateral posterior (LP) nuclei, the pretectal area and the superior and inferior colliculi. Overall, the contribution of GABA to the MPTA from the mesolimbic zone is modest.

#### 3.3.3 Medulla, cerebellum and spinal cord

**FIGURE 7:**
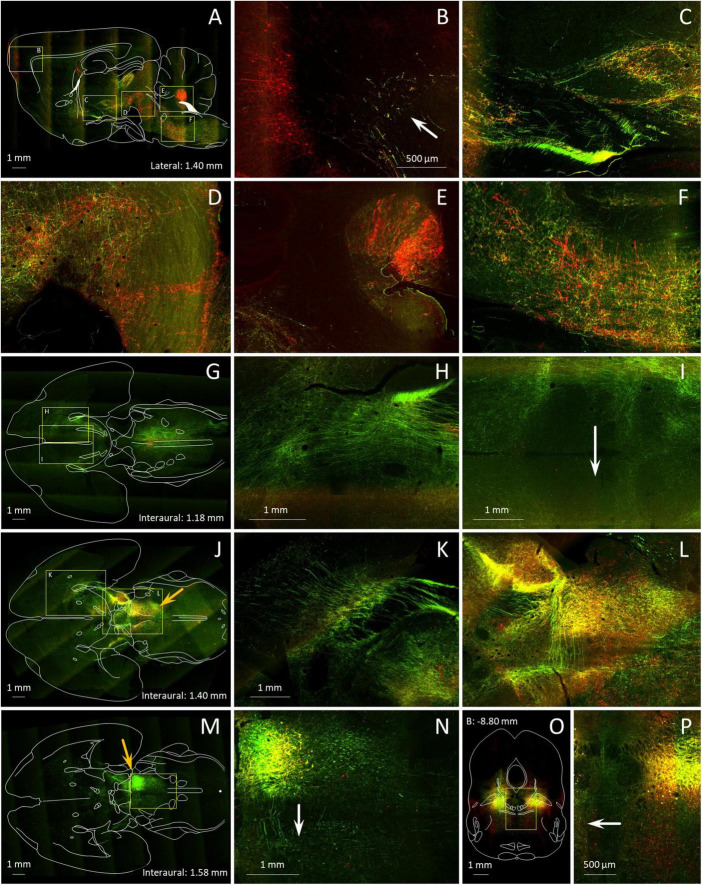
Axonal trajectories of MPTA projection neurons and distant neurons that project into the MPTA. **(A–P)** A mixture of 2 tracers, the anterograde tracer AAV8-eGFP (green) and the retrograde tracer AAVrg-mCherry (red) was microinjected into the MPTA unilaterally **(A–N)**, or bilaterally **(O,P)**. Details of the results are shown at higher magnification in locations marked with yellow rectangles. **(A)** Axons of MPTA projection neurons in this parasagittal section (anterograde, green) can be seen ascending as far rostrally as the PFC (arrow in **B**) while pyramidal projection other neurons retrogradely labeled following AAVrg-mCherry microinjection into MPTA (red fluorescence) send axons caudally toward the MPTA **(B–D)**. Other axons of MPTA projection neurons descend into the pons and medulla **(E,F)**, many destined for the spinal cord. Retrogradely labeled neurons in the DCN and the ventral medulla send axons rostrally toward the MPTA **(E,F)**. An outline drawing from the Paxinos and Watson rat brain atlas is superimposed. **(G,J,M)** show horizontal brain sections in sequence, dorsal to ventral, again with outline drawings superimposed. Depth coordinates of each are indicated. The section in **(M)** is cut through the MPTA microinjection site, still visible in **(J)** (orange arrows). In all 3 panels ascending and descending axons can be seen, mostly in the mesolimbic (ventral) stream, extending rostrally all the way into the striatum and frontal cortex (**H,K,L),** but also caudally in the brainstem. Most axons run in pathways ipsilateral to the microinjection site. However, axons of some MPTA projection neurons (green) are clearly visible crossing to the contralateral side, anteriorly in the anterior commissure (**I**, arrow), and further caudally in the ventral tegmental decussation and at the level of the MPTA itself (**L,N**). **(O)** a coronal section through the MPTA in a rat microinjected bilaterally with anterograde (green) and retrograde (red) tracers. The midline area [yellow rectangle in **(O)**], enlarged in **(P)**, highlights decussating axons passing in both directions.

Quite dense retrograde labeling was observed in GABAergic neurons in the DCN, represented by ROIs INTa (interpositus), LAT (lateral, dentate) and LATpc (parvocellular part of lateral nucleus). Percentages of double-labeling here rivaled those of the cerebral cortex although due to size, overall numbers were far smaller (Table 3, Figures 5K,L, 7E). Most such cells occurred ipsilaterally, but this varied from animal to animal, probably reflecting the location of the microinjections with respect to the level of decussation of the superior cerebellar peduncle. Previous research with more rostral targeting found most DCN projection neurons contralaterally (Sukhotinsky et al., 2007). A felt-work of traversing axons was observed in the reticular core of the medulla, but there were no compact bundles or obvious clusters of double-labeled neuronal somata (Figure 5M). As noted, no GABAergic projection neurons were observed in the cervical SC (Figure 5N).

### 3.4 Reciprocal connectivity of GABAergic neurons within the MPTA

**FIGURE 8:**
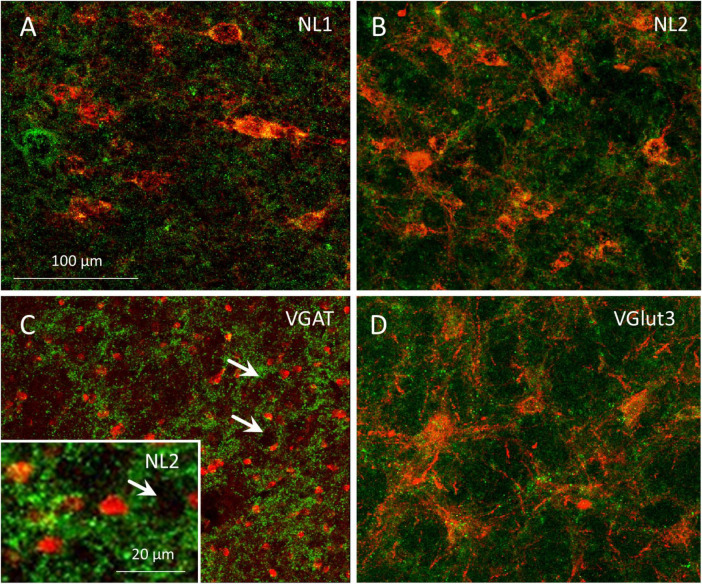
Axon terminals of GABAergic and glutamatergic projection neurons associate with resident cells of the MPTA. **(A–D)** Single-plane confocal images of MPTA effector-neurons, visualized by mCherry expression following AAV8-mCherry microinjection into the MPTA with enhancement using anti-mCherry immuno-labeling (large red neurons). Afferent axons and terminals in **(A)** show NL1-IR (green fluorescence) marking glutamatergic endings in association with effector-neurons in the MPTA. **(B)** NL2-IR (green) marks GABAergic and/or glycinergic endings, adjacent to MPTA effector-neurons. **(D)** VGlut3-IR (green) marking glutamatergic endings in association with MPTA effector-neurons. **(C)** A confocal image of VGAT-IR (green) marking GABAergic fiber endings in the vicinity of MPTA δ-cells, immuno-labeled with an anti-GABA_*A*_δ-R antibody (red). The inset is similar, but using anti-NL2-IR. Note the small size of δ-cells compared to effector-neurons. Rings of GABAergic terminals can be seen surrounding medium-to-large neurons that are not immune-labeled (white arrows). The scale-bar in **(A)** refers to panels **(A–D).**

In 4 rats we searched for GABAergic neurons within the boundaries of the MPTA that sent an axonal projection to the MPTA on the opposite side. All 4 received unilateral AAVrg-mCherry and icv colchicine to enhance the visibility of GAD67, and 2 additionally received a colchicine microinjection into the MPTA. In a 500 × 500 μm counting frame centered on the MPTA we counted a total of 75 commissural neurons in these 4 rats (red, 75/mm^2^), 51 of which were also GABAergic (green, 68%). The 2 rats with microinjected colchicine contributed 52 of the 75 commissural neurons and 39 of the 51 of the GABAergic ones, suggesting that colchicine indeed enhances GABA visibility. The commissural neurons that were visualized were relatively large (8–29 μm, mean 16.4 ± 5.3 μm; averaging long and short diameters), with most bearing prominent dendrites. By these criteria few if any would have been δ-cells that are round or elliptical and mostly measure 5–7 μm across (Figure 8C).

### 3.5 GABAergic, glycinergic and glutamatergic axon terminals within the MPTA

Documentation of pre-terminal axons and synaptic terminals in the MPTA confirmed our conclusions about GABAergic input from afar, identified based on retrograde labeling. Additionally, these images provided information on the populations of MPTA-resident neurons that receive the input. Specifically, essentially all cell bodies and proximal dendrites of MPTA effector-neurons, identified by AAV8-mCherry expression (Figure 8, red), had GABAergic, glycinergic and glutamatergic axon terminals nearby (Figures 8A,B,D; green). In addition, Figure 8C (arrows) shows distinct rings of GABAergic, VGAT-IR and NL2-IR terminals encircling medium-to-large MPTA neurons. Because these were not exposed to AAV8-mCherry we cannot be sure that any give neuron is an effector. However, from previous work in which this AAV was used, we know based size, shape and NeuN immunoreactivity that about half of these would likely have been effector-neurons and the other half neuronal types other than effector-neurons (Baron et al., 2022; Baron et al., 2025).

Also shown, in Figure 8C are numerous much smaller δ-cells, identified by GABA_*A*_δ-R-IR (red). The δ-cells, like the much larger effector- and unidentified MPTA neurons, are embedded among incoming GABAergic axon terminals (green) as well as among glycinergic and glutamatergic terminals (not shown). These terminals, however, do not encircle δ-cells in the same embrace with which they encircle the larger neurons in the field (Figure 8C). This suggests close apposition, but not necessarily synaptic contact. The δ-cells might well be exposed to GABA and other neurotransmitters in the interstitial medium rather than within the synaptic cleft (see Discussion). It is important to note that NL1 and NL2 are antigens expressed by the postsynaptic neuron. Hence they are unlikely to represent free axon endings, or axons *en passage.* On the other hand, without corresponding ultrastructural images we cannot be sure that they indeed represent synaptic terminals of projection neurons.

### 3.6 Trajectory of axonal pathways of MPTA projection neurons

**FIGURE 9:**
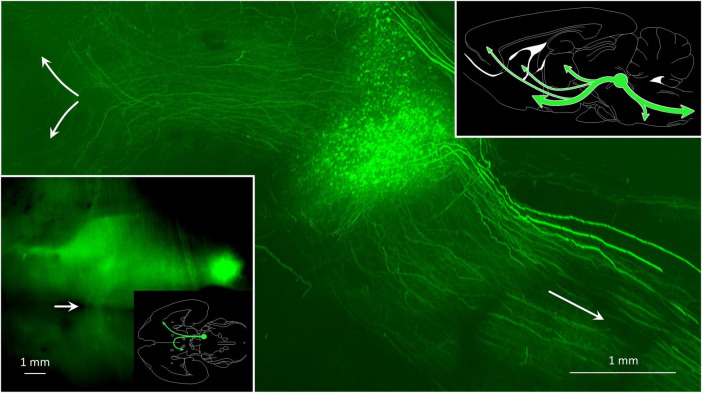
Some MPTA projection neurons maintain axons that ascend to synaptic targets in the forebrain, while for others axons descend toward terminations in the brainstem and spinal cord. The main image is a parasagittal light-sheet image of a rat brain rendered transparent using CLARITY. The anterograde tracer AAV8-eGFP was microinjected into the MPTA (green fluorescent cell cluster), indicated as a green circle in the summary sketch (upper right). The few extraordinarily thick axons on the dorsum of the pons and medulla likely reflect an optical artifact. The inset (lower left) shows a horizontal image of the same brain, oriented as in the adjacent outline drawing which also shows the location of the MPTA. Note the bundle of ascending axons that cross the midline with ventral tegmental decussation (white arrow) and proceed caudally on the contralateral side.

This, and our prior studies of MPTA connectivity, rested mostly on serial/coronal sections. This plane-of-section is well suited for identifying nuclear targets of MPTA projection neurons and the location of neuronal groups that send axons into the MPTA. But it is less suited for visualizing the trajectory of ascending and descending axonal pathways, mostly cut transversely in coronal sections (Baron et al., 2022; Sukhotinsky et al., 2016). To better visualize the trajectories of these pathways brains of AAV8-eGFP microinjected rats were examined in transparent brain whole-mounts (Figure 9). In addition, we combined an anterograde and a retrograde tracer in single MPTA microinjections, and cut brains in the sagittal and horizontal planes (Figure 7). Note, however, that the tracers used, AAV8-eGFP (anterograde) and AAVrg-mCherry (retrograde), are not selective for GABAergic neurons.

Figure 9 shows a whole-mount preparation in which the MPTA was microinjected with AAV8-eGFP. Large numbers of axons can be seen ascending from the injection site and then turning either ventrally to enter the mesolimbic pathway, or dorsally on their way to the iTh (main image, upper left, green). Other axons descend toward the medulla and the spinal cord (main image, lower right, green). As the MPTA does not provide any input to the DCN, no fibers can be seen exiting the MPTA to enter the superior cerebellar peduncle and the cerebellum. The DCN, on the other hand, sends heavy GABAergic input into the MPTA. These projection neurons, and some fibers, are marked with the red retrograde tracer in Figures 7A,E. Prior research using double retrograde tracing established that projection neurons resident in the MPTA show very little collateralization (Goldenberg et al., 2018; Lellouche et al., 2020). Thus, most of the ascending and descending axons illustrated in Figures 7, 9 must originate in different neurons within the MPTA, as do those that terminate in different nuclear targets within the forebrain. A summary of the major ascending and descending pathways of the MPTA are shown in the sketch in Figure 9 (upper right).

#### 3.6.1 Trajectory of fiber pathways rostral to the MPTA

Anterograde tracing in sagittal sections nicely visualizes the 3 primary ascending pathways inferred from this and our prior studies (Figures 7A–D). The first, a ventral stream, tracks past the VTA and ZI, and follows the medial forebrain bundle to the BF with some fibers turning laterally to enter the amygdaloid complex (Figures 7A,C,D). A small contingent of these fibers ascends in the cerebral peduncle to end in the PFC (Figure 7A; Sukhotinsky et al., 2007). We confirmed this here with the observation of a small number of retrogradely labeled neurons in the ipsilateral MPTA following injection of FG into the PFC in two rats. The second pathway, a dorsal stream, passes through the mesopontine tegmentum and pretectal area to end in the intralaminar thalamus (iTh) (Figure 7A). Finally, some fibers belonging to both streams arc dorsally to enter the genu of the corpus striatum (Figures 7B,H,K; Baron and Devor, 2023). Labeled ventral stream axons traversing the mesolimbic zone were about half green (anterograde) meaning that their cell soma was in the MPTA. The other half were red (retrograde) indicating distant cell somata with axons projecting into the MPTA (Figures 7A,C,D).

#### 3.6.2 Trajectory of fiber pathways caudal to the MPTA

As in the forebrain, axons traversing the pons and the medulla contained roughly equal numbers of axons descending from the MPTA (anterograde, green) as axons ascending from the spinal cord and caudal hindbrain (retrograde, red). But in contrast to the compact axon bundles of the forebrain, these were disordered, particularly the ascending ones (red), appearing to criss-cross the reticular formation (Figures 7A,E,F,G). The DCN sends numerous ascending axons, probably all GABAergic, as input to the MPTA, but receives no input back from the MPTA (red in Figures 7A,E).

#### 3.6.3 Commissural pathways of MPTA projection neurons

**FIGURE 10:**
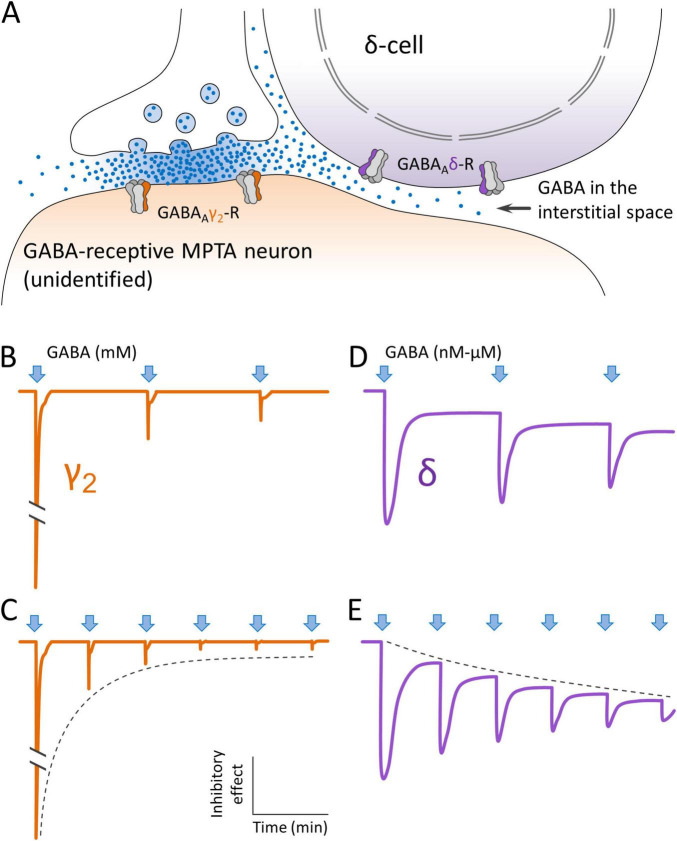
Concept of how GABAergic projection neurons might deliver GABA to extrasynaptic GABA_*A*_ receptors on MPTA δ-cells. **(A)** GABA is released from a synaptic terminal, accessing GABA_*A*_γ2-Rs on the postsynaptic membrane of a GABA-receptive neuron that is neither a δ-cell, nor an effector-neuron. Some of the GABA leaks out of the synaptic cleft and accesses extrasynaptic GABA_*A*_δ-Rs on a nearby MPTA δ-cell, by spillover. **(B,C)** The initial (phasic) inhibitory effect of GABA release on GABA_*A*_γ2-Rs (arrows) is very large. But due to receptor adaptation and desensitization it declines rapidly with repeated impulses invading the presynaptic terminal, even though GABA continues to be released into the cleft (dashed line). **(D,E)** Extrasynaptic GABA_*A*_δ-Rs behave differently. Their initial phasic response is smaller than that of GABA_*A*_γ2-Rs, but they retain a (tonic) inhibitory effect for as long as GABA is present. This tonic inhibitory effect likely increases with repeated impulse activity in proportion to the concentration of GABA in the adjacent interstitial space (dashed line). Note that sketches **(B–E)** illustrate a concept. They are not traces from actual electrophysiological experiments.

Axons passing from the MPTA on one side to the other side take 3 alternative routes. A minority take a direct route, crossing the midline at the level of the MPTA itself. This can be seen in both horizontal (Figures 7M,N) and coronal sections (Figures 7O,P). A larger contingent of crossing fibers travel ∼5 mm rostrally through the mesolimbic zone in the ventral stream of ascending fibers, and turns 90° medially to cross the midline in the ventral tegmental decussation. On the contralateral side some of these turn again by 90°, forming a U-shaped trajectory and return caudally within the contralateral ventral mesolimbic pathway (Figure 10 inset). Others continue rostrally on the contralateral side (Figures 7J,L). Finally, some fibers continue rostrally on the side of the microinjection and cross as a part of the anterior commissure (arrow in Figure 7I).

## 4 Discussion

Transitioning between wakefulness and unconsciousness occurs in concussion, epilepsy and other brain pathologies, but it is also a part of normal life. Examples include natural sleep and syncope (fainting), and in some species tonic immobility, torpor and hibernation. In each case immobility is accompanied by high δ-band power EEG, suggesting loss-of-consciousness (Carli, 1969; Carli and Farabollini, 2022). The discovery that a similar brain-state transition can be induced by exposure of a small population of mesopontine neurons to low concentrations of GABA_*A*_-R agonists suggests the operation of a mechanism common to them all, and motivates consideration of how endogenous GABA might access the MPTA to initiate transitioning in non-experimental situations. Although neurons capable of releasing GABA in the brainstem have been well studied, we are not aware of prior reports focusing on the MPTA (Chen et al., 2019; Ford et al., 1995; Jones, 1995; Jones et al., 1991; Sapin et al., 2009; Vertes and Kocsis, 1997). We found three main contributors of GABA to the MPTA: rostral neocortex, the mesolimbic continuum and two hindbrain sites, all with a strong ipsilateral predominance. Deep laminae of the frontal cortex are the predominant contributors. Interestingly, nearly all of the regions identified here have been implicated previously in wakefulness and arousal.

It is inevitable, even using small microinjections, that some retrograde tracer will fall outside of the intended target. Thus, theoretically, many or all of the neurons registered as terminating within the MPTA might in fact have terminated outside. In a *post hoc* analysis we ruled this out in two ways. First, for each ROI we asked whether there is a correlation between the density of retrogradely labeled neurons counted and the area of tracer inside vs. outside the target rectangle. Combining these data yielded a statistically significant correlation between cell count and tracer area within the MPTA frame (*p* < 0.001), but not for tracer areas outside the MPTA frame (*p* = 0.90). Second, we compared the rat with largest overall microinjection area (#AR33, overall 3.18 mm^2^, only 34.6% of which was inside the MPTA) with the rat with the smallest (#AR13, 1.24 mm^2^, 76.4% of which was inside the MPTA; Figure 1). The density of retrograde labeling (neurons/mm^2^) for injection areas inside the MPTA was very similar in 14 of the 15 ROIs available in both animals (*R*^2^ = 0.811, *p* = 0.01 (Figure 4D). This indicates that retrograde labeling could not have been due to trace located exclusively outside of the MPTA. In only one ROI, PRh, was there a substantial difference in neuronal density, 56.1/mm^2^ in #AR13 vs. 573.1/mm^2^ in #AR33. This suggests that the PRh cortex sends most of its projections to the cuneate, a nucleus residing dorsal and lateral to the MPTA, that was exposed to tracer in rat #AR33, but not in rat #AR13. Both calculations rule out the possibility that the GABA projections described are all, or mostly, directed to areas outside of the MPTA. To be sure, there is every reason to believe that regions outside the MPTA also receive GABAergic input, including from ROIs that provide such input to the MPTA. Our experimental question, however, concerned sources that deliver GABA to the MPTA, irrespective of whether they also deliver GABA to adjacent areas outside.

### 4.1 Marking projection neurons as GABAergic

A potential concern with tracer studies is differential uptake, transport and visibility of the tracer, factors that in principle could yield labeling of idiosyncratic subsets of projection neurons. This in mind, we employed two retrograde tracers with quite different characteristics, FG and AAVrg-mCherry. FG, like cholera toxin-b chain (CTB) used as a retrograde tracer in some of our previous studies, is presumed to enter axon terminals non-selectively, by pinocytosis from the bulk extracellular medium. There it loads onto microtubules and trafficks to the cell soma where it is visualized within transport vesicles. Entry of AAV-based tracers is more complicated. This additionally involves preferential binding to serotype-specific membrane epitopes and, after retrograde transport, accessing transcription and translation machinery where mCherry is expressed in a promoter-dependent manner (Murlidharan et al., 2014; Nassi et al., 2015). Despite these differences the location of projecting neurons revealed using FG and AAVrg-mCherry as tracers was very similar (Figures 4B,C), and largely the same as those we found previously using CTB (Sukhotinsky et al., 2007). AAVrg-mCherry proved to be the more efficient tracer. This is probably because of the continued accumulation of mCherry over weeks *in situ*. Visualization of GAD67, a reliable marker of GABAergic neurons, was enhanced using colchicine (Ribak et al., 1978). Additionally, we used VGAT and NL2 as supplemental markers of GABAergic somata and terminals. We conclude that GABA input to the MPTA is abundant and derives mostly from distant GABAergic neurons, mostly in deep layers of the frontal cortex, with the caveat that immuno-labeling in the tegmentum may have been suboptimal.

### 4.2 Dual action of GABA release in the MPTA

#### 4.2.1 Cellular and molecular targets of GABA within the MPTA

Upon synaptic release GABA rapidly reaches high concentrations within the synaptic cleft, in the low millimolar range. Such concentrations open synaptic GABA_*A*_γ2-Rs. Once activated, however, the resulting synaptic current adapts to zero within a second or two, despite the continued presence of the agonist. Moreover, following GABA washout, an additional period of time goes by before the desensitized receptor recovers responsiveness. Because of the highly phasic nature of the resulting neurotransmission, the synaptic GABA_*A*_γ2-R is unlikely to be the driver of anesthesia, a state that persists for hours and days with continued drug delivery (Baron et al., 2025). Rather, a tonic, non-adapting inhibitory process likely mediates anesthesia, and by extension also endogenous instances of LOC such as natural sleep, hibernation etc. These are likely mediated by a second class of GABA_*A*_-R, receptors that reside extrasynaptically, are highly sensitive to GABA and its endogenous congeners (below), are tonic, and mostly incorporate the δ subunit in place of γ2, with other subunits also playing a role (Ahring et al., 2016; Barberis et al., 2007; Baron and Devor, 2024; Bright et al., 2011; Stórustovu and Ebert, 2006). Evidence that this general conclusion applies also to the MPTA is summarized by Baron et al. (2025).

GABA_*A*_δ-Rs, in contrast to synaptic GABA_*A*_γ2-Rs, respond to mid-nanomolar concentrations of GABA and the resulting Cl^–^ current appears to persist for as long as the agonist is present. This makes the GABA_*A*_δ-R the prime candidate mediator of sustained brain-state transitions driven by GABAergic anesthetics, and by endogenous circulating and interstitial molecules such as neurosteroids and other somnogens (e.g., adenosine and taurine (Baron and Devor, 2024; Baron et al., 2025; Hemmings et al., 2019). We propose that this is also the case for GABA itself. GABA is known to be present in the narrow spaces interstitial between neurons and glia in the CNS, entering these spaces from the ventricular cerebrospinal fluid (CSF), the intracerebral vasculature and released within brain parenchyma from free axon endings and from astroglia. Concentrations of GABA in the CSF due to these sources are low, are expected to be quite stable over time, and not likely to be regionally specific. But in addition to these there is an important additional source of extracellular GABA, one that may well vary rapidly and be regionally specific. This is leak from the synaptic cleft with spillover into the interstitial space (Bazargani and Attwell, 2017; Lee et al., 2010; Wei et al., 2003; Figure 10).

The spillover mechanism, in principle, can allow the concentration of interstitial GABA to which extrasynaptic GABA_*A*_δ-Rs are exposed to be subject to regulation on a moment-to-moment basis by control of the discharge pattern of afferent GABAergic input neurons. For example, intense and prolonged synaptic input to the MPTA from distant sources would raise the concentration of interstitial GABA to levels still low, but high enough to strongly activate extrasynaptic GABA_*A*_δ-Rs on MPTA δ-cells. A result would be to drive the individual into a deep sleep. By the same token, moderate discharge of GABAergic axon terminals in the MPTA would yield moderate GABA spillover from the synaptic cleft generating a lighter, more labile sleep state permitting arousal, for example, by stimuli such as a baby’s cry. In both scenarios the synaptic action on GABA-receptive neurons would necessarily be mediated by GABA_*A*_δ-Rs on δ-cells (Figure 10). An action on neurons that express GABA_*A*_γ2-Rs is largely excluded both because of their rapid adaptation and desensitization, and because these receptors do not respond to the low concentrations of GABA present outside of the synaptic cleft due to spillover. GABA-receptive neurons in the MPTA that express GABA_*A*_γ2-Rs probably act in the awake state, making essential contributions to the complex, phasic, local-circuit computations executed by a conscious brain. These would presumably be executed using intermittent spikes, spike bursts, or other patterns of activity that minimize receptor desensitization and GABA spillover.

Overall, by close regulation of discharge patterning, the brain should be able to recruit GABAergic inputs to the MPTA in a dual manner. Housekeeping functions and functions of wakefulness would mostly operate synaptically using (phasic) GABA_*A*_γ2-Rs. Transitioning to unconsciousness would occur with a regulated shift to the more tonic discharge patterns that would foster accumulation of GABA locally at the relatively low concentrations that are the sole domain of extrasynaptic GABA_*A*_δ-Rs, yielding light (arousable) forms of sleep, through deep sleep, torpor and hibernation. Specific evidence of the spillover mechanism in the MPTA is the observation that pharmacological block of synaptic reuptake of GABA within the MPTA with tiagabine, a process expected to increase spillover, is pro-anesthetic (Baron et al., 2025; Figure 10).

#### 4.2.2 Interaction of MPTA δ-cells and effector-neurons

Immunolabeling previously identified δ-cells as the sole carriers of extrasynaptic GABA_*A*_δ-Rs in the MPTA (Baron et al., 2022). This establishes their likely role as the cellular target of GABAergic general anesthetics, interstitial GABA, neurosteroids etc. Here, using NL2 and VGAT as markers, we documented abundant GABAergic terminations amongst these cells, effector-neurons and also unidentified medium to large diameter MPTA neurons. Ultrastructural analysis will be required to determine which of the three actually receive GABAergic input via synapses. However, as relatively few δ-cells or effector-neurons express GABA_*A*_γ_2_-Rs, and in any event effector-neurons need to be excited to induce LOC, direct modulation via GABAergic synapses is unlikely. Rather, we suspect that synaptic GABAergic input to the MPTA is probably targeted to GABA_*A*_γ_2_-Rs expressed by the unidentified neuronal population, making these a key source of spillover. Indeed, these are the cells most intimately embraced by labeled axon terminals (Figure 8C). One therefore needs to know how δ-cells, responding to synaptic spillover, might interact with effector-neurons to induce LOC.

A hint at answering this question is the observation that nearly all effector-neurons are closely apposed to one or more δ-cells (Baron et al., 2025). This suggests that the activity of effector-neurons is modulated by activity in δ-cells by a mechanism not yet defined, perhaps paracrine or electrical (Figure 10). We have predicted that this putative mechanism is inhibitory. Specifically, we suppose that during wakefulness δ-cells fire spontaneously, maintaining effector-neurons in silence by a mechanism not yet defined. Suppression of this activity with GABAergic anesthetics, interstitial GABA, neurosteroids etc. would lead to excitation of effector-neurons (by disinhibition) and hence the induction of sedation and LOC (Baron and Devor, 2024; Baron et al., 2025; Devor et al., 2016). Alternative, or additional mechanisms are also available to drive effector-neurons. For example, the ample glutamatergic input to the MPTA could provide direct excitation of MPTA effector-neurons and hence also contribute to sedation, sleep, torpor etc. We do not know if inputs to the MPTA from individual neurons might release both GABA and glutamate, although such co-transmission has been reported elsewhere in the brainstem (O’Brien and Berger, 1999; Vaaga et al., 2014).

### 4.3 Laterality and reciprocity

GABAergic input to the MPTA is strongly ipsilateral with a preference of roughly 6:1 across the 19 ROIs. Likewise, overall ascending and descending projections of MPTA neurons to their main nuclear targets are also preferentially ipsilateral, albeit with a ratio of about 2:1 rather than 6:1 (Goldenberg et al., 2018; Lellouche et al., 2020). Nonetheless, functional switching of the cortical EEG signature upon unilateral MPTA microinjection of GABAergics is bilateral and symmetrical (Avigdor et al., 2021). The decussation of MPTA projection neurons may account for this, or perhaps corico-cortical connectivity.

As for reciprocity, although the 19 ROIs were selected based on axonal projections to the MPTA irrespective of axonal inputs, many of them also receive input from the MPTA, particularly ROIs in the mesolimbic zone. Other regions showed little or no reciprocal connectivity. The DCN, for example, send substantial GABAergic input into the MPTA, but receive no input from the MPTA. Conversely, the iTh receives input from the MPTA, but does not reciprocate with GABA output (Baron et al., 2022; Lellouche et al., 2020; Sukhotinsky et al., 2007). The cerebral cortex is a special case. On the one hand, it is the most prominent contributor of GABAergic input to the MPTA while receiving only minimal *direct* input from the MPTA, focused in the PFC. On the other hand, the cortex does receive abundant *indirect* MPTA input, relayed through the iTh, ZI and BF. Indeed, the MPTA stands out for its ability to rapidly and reversibly switch the cortical EEG signature from the wake state to oblivion (Avigdor et al., 2021).

### 4.4 Functional roles of the major sources of GABAergic input

#### 4.4.1 GABAergic inputs from the neocortex

The frontal neocortex is widely cited as the seat of conscious arousal, with many high-order functions attributed to it including sensory perception, planning, decision-making, attention and emotions, especially in primates (Friedman and Robbins, 2022; Libedinsky and Livingstone, 2011; Mashour et al., 2022, but see Baron and Devor, 2022). One should recall, however, that homology between the primate PFC and the rodent rostral pole is uncertain (Laubach et al., 2018). None of the ROIs fell within caudal parietal or occipital regions, an observation that may speak to a current controversy concerning the relative roles of rostral vs. more caudal regions of the cortex in higher cognitive function (Boly et al., 2017). The more primitive paleo- and archi-cortex (piriform lobe and hippocampal formation) appear to contribute minimally if at all. It is notable that much of the MPTA’s GABAergic input originates in association neocortex. Perhaps this explains why sustained cognitive effort, or the dull repetitive input of a boring lecture, may lead to (spillover) drowsiness.

#### 4.4.2 GABAergic inputs from the mesolimbic zone

Like the medulla this region is deeply involved with homeostasis. But unlike the medulla, it operates via neuroendocrine regulation rather than the autonomic nervous system. It also mediates higher brain functions including emotions (e.g., attachment, fear, pleasure) and motivated behaviors (e.g., feeding, drinking and reproduction). For example, dopaminergic neurons of the VTA (and to a lesser degree the SN), and more rostral zones including LH, POA and nucleus accumbens, are important regulators of reward and reinforcing properties of food, water and sexual experience. All of these functions are associated with arousal, as is a major ascending extra-thalamic pain pathway that originates in the spinal substantia gelatinosa, relays through the parabrachial area and mesolimbic Ce, ending in the cerebral cortex. Noxious stimuli (e.g., tail-pinch) and electrical stimulation anywhere along ascending pain pathways lighten anesthesia transiently (Fields and Basbaum, 1999; Gauriau and Bernard, 2002; Leknes and Tracey, 2008; Pert, 1997; Stuber and Wise, 2016). Interestingly, the mesolimbic zone is also intimately related to sleep, containing the VLPO and associated sleep nuclei such as the supraoptic nucleus, the perifornical orexinergic neurons of the LH and the histaminergic TMN. Perhaps serving both arousal and somnogenic functions the MPTA relays to the cortex through the thalamus (iTh), but also through the BF and the ZI, alternative extra-thalamic pathways.

#### 4.4.3 GABAergic inputs from the cerebellum and medulla

An intriguing aspect of our observations is the link between sensory and motor aspects of GABAergic involvement in MPTA function. The fact that dopamine originating in the SN is a key component of extrapyramidal motor function and also of pleasure and arousal is not immediately intuitive. But it makes sense with respect to the axis of wakefulness and sedation. The link between intense physical effort and the dual feelings of elation and exhaustion may be another case in point. A counterintuitive example is the location of the MPTA along the ascending course of the superior cerebellar peduncle from which it receives plentiful GABAergic input from the DCN (Shammah-Lagnado et al., 1983; Sukhotinsky et al., 2005). Classically the cerebellum has been considered a structure controlling movement, although the disproportionally large cerebellum of elephants, animals not know for feline agility, has long brought this idea into question. But what link might there be to wakefulness and sedation? An answer might lie in the various higher-level cognitive roles added in recent years to the repertoire of the cerebellum (Callu et al., 2013; Sathyamurthy et al., 2020).

Finally, the RVM has multiple roles associated with MPTA function. Among these are sleep atonia and descending pain inhibition directed to the trigeminal and spinal dorsal horn. Indeed, descending projections to the RVM from the MPTA are roughly as prominent as those from the more well-studied ventrolateral periaqueductal gray (vlPAG) (Fields and Basbaum, 1999; Lefler et al., 2008; Mason, 2001; Sukhotinsky et al., 2005). Minimal GABAergic input to the MPTA was found at mid-levels of the medulla, and its caudal extent. This is surprizing in light of the importance of this part of the brainstem for homeostasis, with syncope a frequent consequence of dysregulation (Janig, 2022). MPTA lesions do affect syncope in the presence of hypercapnia, but they do not have major impact on systemic blood pressure or thermoregulation, and they do not cause LOC (Avigdor et al., 2021; Meiri et al., 2016; Minert et al., 2020).

### 5 Summary and conclusion

The MPTA is a singular locus, the only one reported to date at which surgical anesthesia can be induced by direct delivery of GABAergic anesthetic agents by microinjection. A pro-anesthetic effect is obtained using concentrations actually present in the brain upon systemic delivery. Correspondingly, lesioning the MPTA reduces the efficacy of GABAergic agents administered systemically. These observations suggest that MPTA effector-neurons play a key role in dedicated circuitry underlying anesthetic loss-of-consciousness. Hallmark indicators, including EEG synchronization and loss of spinal reflexes, point to cortical and spinal actions which, as noted, could not be due to translocation of effector molecules from the site of microinjection to the cortex or cord (Baron and Devor, 2023; Baron et al., 2025; Minert et al., 2020).

The fact that the idiosyncratic symptoms of anesthesia: atonia, analgesia, amnesia and LOC, also characterize physiological instances of LOC including syncope, concussion, epilepsy, tonic-inhibition, natural sleep, hibernation and others, suggests that all may share a common regulatory mechanism. Our observations suggest that the MPTA is an important node in the evolutionarily adaptive circuitry dedicated to effecting the brain-state transitions associated with LOC. They also support the notion that exogenous GABAergic anesthetics act by substituting for an endogenous neurotransmitter to co-opt this circuitry, the locus of this substitution being, at least in part, extrasynaptic GABA_*A*_δ-Rs expressed by MPTA δ-cells. The main endogenous neurotransmitter is presumably GABA, sourced largely by spillover from GABAergic synapses of projection neurons located in the various ROIs identified in this study. Given its small size, the large number of such neurons that send axons into the MPTA is impressive. Equally broad swaths of brain contain no such neurons (Results sections 3.3.1, 3.3.2, 3.3.3 for cortex, subcortical forebrain and hindbrain, respectively). Other candidate agonists that might play subtly different functional roles such as mood modulation, include neurosteroids, somnogens and related diffusible agents (Baron and Devor, 2024). Further elucidation of the circuitry underlying LOC holds the potential of intuiting why the individual components of anesthesia are so tightly bound together, and why motor control, sensory experience and awareness all collapse in unison, while complex housekeeping functions of the brain and at least the early phases of sensory computation, persist.

## Data Availability

Data from this study will be provided to qualified investigators upon reasonable request. Requests to access the datasets should be directed to MD, marshlu@mail.huji.ac.il.
